# Barriers and facilitators of compliance with infection prevention and control measures during the COVID-19 pandemic in health facilities in Kampala city, Uganda

**DOI:** 10.1371/journal.pgph.0004021

**Published:** 2024-12-09

**Authors:** Mitima Jean-Marie Limenyande, John Bosco Isunju, David Musoke

**Affiliations:** Makerere University School of Public Health, Kampala, Uganda; Child Health Research Foundation, BANGLADESH

## Abstract

During the COVID-19 pandemic, Infection Prevention and Control (IPC) was crucial to reduce the spread of the virus in health facilities. This study explored the barriers and facilitators of IPC compliance among healthcare workers (HCWs) during the COVID-19 pandemic in Kampala City, Uganda. Key informant interviews were conducted with 14 participants in 12 health facilities located in Nakawa division, Kampala City. Of these facilities, 3 were government-owned, and 9 were private not-for-profit. Each health facility’s participant was either the IPC focal person or a HCW knowledgeable about the IPC measures implemented there. Transcripts were coded using a newly generated codebook in Atlas.ti version 9, and thematic analysis was carried out to analyze the study findings. Participants identified the fear of contracting the virus as one of the primary facilitators for IPC compliance among HCWs during the COVID-19 pandemic. They explained that the sustainability of IPC measures in health facilities was partly due to HCWs’ belief that they served as role models in the community for observing and implementing health-related behaviors, especially regarding COVID-19. Among the barriers, participants mentioned that not all HCWs got the opportunity to be trained on COVID-19 IPC. Only HCWs working in high-risk departments for COVID-19 such as triage or Intensive Care Units were prioritized. However, regardless of the department, all HCWs were exposed to potential COVID-19 patients, and the high workload led them to sometimes skip the required IPC measures. This study found that addressing the spread of COVID-19 among HCWs necessitated a comprehensive approach to IPC in health facilities. This approach should encompass capacity building, as well as provision of necessary supplies. In addition, HCWs, the hospital management and government have a role to play to ensure that IPC is fully implemented in the case of future related outbreaks.

## Background

The COVID-19 pandemic underscored the urgent need for improved Infection Prevention and Control (IPC) practices in health facilities worldwide, including Uganda [1–4]. With the daily number of new infections reaching several hundreds in the country at the peak of the pandemic, health facilities had to prepare to accommodate the escalating number of COVID-19 patients while ensuring the safety of healthcare workers (HCWs) [5,6]. Meanwhile, IPC has been a significant concern in health facilities for centuries, with infections being the leading causes of patient mortality before the antibiotic era [7]. Despite advancements in IPC, infections continue to pose a major challenge in health facilities, resulting in numerous deaths. Approximately 7 out of 100 patients in developed countries and 15 out of 100 patients in developing countries acquire infections during their stay in acute care units [7,8]. Even among HCWs, many get infected while on duty–and this gets worse during epidemics [9]. Apart from the loss of human lives, the direct and indirect costs of inadequate IPC implementation in health facilities amount to billions of dollars, both in developed and developing countries [10,11].

IPC programs should not only receive attention during outbreaks [12]. Moreover, there are no predefined infections to cover in an IPC program, as each country defines its priorities based on the local context [8,13,14]. Unfortunately, in many low- and middle-income countries, adequate implementation of IPC measures begins only when outbreaks are declared [15]. For example, in Africa, numerous countries lack functional IPC programs, rendering their health systems vulnerable to health emergencies [14,16]. This lack of preparedness has a high-cost implication during new outbreaks as most IPC measures are implemented hurriedly, using approaches that do not guarantee sustainability after the event [17]. Consequently, this unpreparedness poses a significant risk for HCWs and contributes to the spread of infection to patients, caregivers, colleagues, and communities outside the hospitals [18,19].

In Uganda, the Ministry of Health has developed IPC guidelines for health facilities in recent years, along with training manuals to ensure IPC implementation in accordance with country standards [20]. Specific guidelines were also formulated to meet IPC requirements during the COVID-19 pandemic as the virus spread across the country. This was crucial, given the potential devastating impact on service delivery in a country where the ratio of HCWs to the population served is inadequate [21]. Despite frequent reports of scarcity of Personal Protective Equipment (PPE) and other IPC essentials such as hand sanitizer for hygiene in various health facilities across the country, HCWs remained active in providing care to COVID-19 patients. However, this unsafe working environment raised concerns about HCWs’ safety, necessitating urgent solutions. It was essential to understand what motivated HCWs to adhere to appropriate IPC precautions while delivering care in such challenging circumstances. Therefore, the aim of this study was to explore the barriers and facilitators of IPC compliance during the COVID-19 pandemic among HCWs providing care to patients in health facilities in Kampala City.

## Methodology

### Study design, area, and selection of participants

This qualitative study utilized key informant interviews (KIIs) to explore participants’ perspectives on barriers and facilitators of IPC compliance [22,23]. It was conducted across 12 health facilities situated in Nakawa Division, Kampala city, Uganda. Of these, three were government-owned facilities, while nine were private not-for-profit (PNFP). The facilities comprised one hospital, one Health Centre IV, three Health Centre IIIs, and seven Health Centre IIs. Details regarding the selection of health facilities and descriptions of the services offered at each level have been provided in another paper [24]. At each health facility visited, interviews were conducted with the IPC focal person or a HCW knowledgeable about IPC measures implemented there. Additionally, in the referral hospital, two additional interviews were conducted: one with the in-charge of emergency services and another with the in-charge of the intensive care unit. These extra interviews were necessary due to the size of the health facility and the likelihood of COVID-19 patients accessing these services during the pandemic. The 14 key informants were sufficient to achieve thematic saturation for the study as also established elsewhere [25].

### Data collection

The data collection was carried out by two Public Health Officers with experience in qualitative data collection, from 29 March 2022 to 5 May 2022. The KII guide underwent initial review by the research team to ensure its cultural, linguistic, and content appropriateness. Comprising 14 questions and probes under certain questions, the guide addressed three major themes: barriers to IPC compliance; motivators for IPC compliance; and adaptive approaches during periods when PPE are unavailable or when hand hygiene cannot be achieved as recommended [26]. Probes covered various aspects including the perceived severity of COVID-19 by HCWs, fears of infecting colleagues or community members including those at home, and the impact of COVID-19 vaccination coverage among HCWs. The interviews were conducted in English at the health facilities and typically lasted between 25 to 45 minutes. Each interview was recorded using a digital voice recorder, and notes were taken throughout the process.

### Data management and analysis

Daily debrief sessions were conducted among the two Public Health Officers focusing on emerging ideas requiring further exploration during subsequent interviews. The collected data was transcribed by an independent researcher and sent back to the interviewer for verification. Subsequently, the transcripts were imported into Atlas.ti version 9 for coding, with newly generated codes. Initially, the first five transcripts were coded by the two Public Health Officers who collected the data to establish a code structure and ensure alignment with participants’ ideas [27]. The remaining transcripts were coded by one the two Public Health Officer. Upon completion of the coding process, code categories were created, and based on response patterns, they were organized into sub-themes under the two major themes. Thematic analysis was then carried out to summarize the study findings [28].

### Ethical consideration

This study received approval from the Makerere University School of Public Health Higher Degrees Research and Ethics Committee. Additionally, all participants provided signed consent forms before being interviewed, following a clear explanation of the study purpose.

## Results

### Characteristics of the participants

Of the 14 participants who were interviewed, 3 were from the hospital and 9 from the other health centers, with 1 participant selected per heath centre. Twelve participants were female, 3 were aged below 30 years, and 5 held the designation of IPC focal person in addition to their other job profiles at the health facility, Table 1.

**Table 1:** Demographic characteristics of participants (N = 14).

Variables	Number (n)	Percentage (%)
**Sex**		
Female	12	85.7
Male	2	14.3
**Age (years)**		
<30	3	21.4
30+	11	78.6
**Designation**		
IPC focal person	5	35.7
Laboratory technician	3	21.4
Nurse in charge	4	28.6
Doctor	2	14.3

### Facilitators of IPC compliance

#### Fear of contracting COVID-19

According to participants, the fear of contracting the virus emerged as a primary driver for IPC compliance during the COVID-19 pandemic. In the initial phases, HCWs were exposed to the realities of COVID-19 through various media channels, witnessing its devastating impact in other affected regions. This heightened awareness and comprehension of the virus’s severity were essential factors in sustaining a high level of compliance among HCWs.

*“They have seen patients dying, they have seen patients suffer, they have seen so many things. So, that has been also the motivator in them because of the fear”* Participant 5, Lab technician.

*“When you see the TV*, *outside the country people are dying seriously because for us here people were dying but outside it was worse*. *So*, *that one also used to motivated basawo [HCWs] to put the mask and do the rest”*, Participant 1, Lab technician.

These platforms were showing people dying from COVID-19 and no other means of dealing with the virus were available for HCWs in health facilities other than IPC measures. Participants reported that throughout the different waves of the pandemic in the country and particularly in Kampala City, the fear of contracting the virus contributed to IPC compliance among HCWs. They explained that HCWs believed that the virus was severe and could have a serious impact on those who are infected. Meanwhile, some participants expressed different opinions regarding how HCWs perceived the severity of the virus; and according to them, HCWs believed that other diseases were more severe than COVID-19. But over time, participants noted that HCWs kept monitoring the trend in the number of new cases and adapted their level of compliance to IPC measures accordingly. When COVID-19 cases and deaths were rising in and around the country, HCWs were more compliant with IPC. The reduction of new cases had influenced their compliance significantly and thus many had become reluctant in observing IPC measures.

*“They were all relaxed unfortunately they got eighteen people [with COVID-19] at ago. um? So that is when they became very, very serious when they heard that. eh! This one has gotten [us]. I think I am next. That is when they became very serious”,* Participant 7, IPC focal person.

#### Social pressure and moral obligation

Participants explained that the sustainability of IPC measures in health facilities was partly due to HCWs’ beliefs that they served as role models in the community regarding the observance and implementation of health-related behaviors, particularly for COVID-19. According to participants, HCWs believed that the community looked to them for reliable information on which IPC measures were appropriate and when they should be implemented. Therefore, HCWs endeavored to set an example not only out of necessity but also because of societal pressure and a sense of moral obligation placed upon them.

“*We couldn’t enter the clinic without a mask. So, for us, we had to put on a mask so [that] we show people that COVID is what, severe. So, we had to show them [the community] since we are putting on a mask, wash hands, and so on”,* Participant 1, Lab technician.

*“To be their healthcare worker you must do the right thing so that they see you*. *Musawo [the healthcare worker] always do that thing why can’t I copy and do the right thing”*, Participant 7, IPC focal person.

Participants reported that HCWs were reluctant to be perceived as individuals disregarding widely advertised health interventions. Consequently, practices such as mask-wearing and sanitizer use were maintained at a certain level in health facilities despite the decrease in cases across the country. Even in public spaces where mask-wearing had dwindled among the community, HCWs sometimes felt like outliers when they continued to wear masks. Moreover, in certain health facilities, participants attributed high levels of IPC compliance to the proactive involvement of health facility in-charges or department heads. Their active engagement encouraged other team members to follow suit and adhere to necessary protocols. Equally, when department heads showed little interest in IPC measures, the compliance of HCWs also declined.

*“When you are in [public, like in] a taxi you can be alone [with a mask] so they look at you as somebody who is still behind [not up to date]. But for us when we are here [at the hospital], we are encouraged to put on [a]mask. But also, us we know that it is important because we still get those patients [with COVID-19]”,* Participant 11, Nurse in charge.

#### Provision of sufficient IPC supportive equipment

Participants believed that the provision of IPC supportive equipment had positively influenced the level of compliance with IPC measures. These supportive equipment mainly included masks, gloves, sanitizers, or readily available water and soap for use while caring for patients.

*“At the beginning, there was sanitizer along the way [in the health facility]. Somehow the problem of gloves came in but at the end of it, we stocked all of them. When they became available, we bought them in bulk so that we don’t have to face the same challenge again”* Participant 7, IPC focal person.

*“We have not seen any stock out*. *Unless*, *we try to do stock takes if we realize we are running out stock out”*, Participant 4, Doctor.

In cases where IPC requirements were available in the health facility, mechanisms had been put in place to ensure collective access. Similarly, the limited PPE and hand hygiene equipment available were evenly distributed among HCWs. To further enhance accessibility, some health facility in-charges assigned specific individuals roles and schedules to refill sanitizer dispensers regularly, ensuring they were always stocked.

*“When they supply the masks, I have a list of all of them. So, when they supply the masks I come and make sure if they are few, I give five-five [masks]. If they are more, I give ten but I check and see that so and so you received, you received, at least this time. And when I am giving them, I give with caution [so] that if this time we have received few, you might have to buy”,* Participant 11, Nurse in charge.

Participants also believed that PPE were expensive and difficult to provide consistently, impacting HCWs’ compliance with IPC measures. The shortage of PPE in health facilities compelled some HCWs to purchase masks, for example, in order to protect themselves. This underscored the notion mentioned by participants that IPC compliance was primarily for self-protection, thus necessitating such additional sacrifices. However, it was observed that in some facilities, limited access to IPC equipment could result from poor communication between department in-charges for requisitioning PPE and store managers tasked with procurement or supply.

*“It is not easy to maintain if you are to follow. [To] buy PPEs all the time, it is not easy because the money which is there is not enough”,* Participant 2, Lab technician.

### Barriers to IPC compliance

#### Inadequate knowledge on IPC

Not all healthcare workers had the opportunity to undergo training specifically tailored to IPC measures for COVID-19. Priority was given to those working in departments with a heightened risk of contact with COVID-19 patients. Consequently, HCWs who did not receive formal training sought information from alternative sources, which occasionally were unreliable.

*“Some [HCWs] have gone to those courses about the use of PPE but others have not got a chance [of] going to attend. But in case they are given a chance too, I think it will improve the use [of PPE]”,* Participant 5, Lab technician.

The trained HCWs were tasked with mentoring their colleagues, but participants found this mechanism lacking in terms of output delivery at the health facility. These HCWs were primarily trained to ensure their safety and that of their patients. However, there was no specific time allocated for them to train other HCWs. This highlighted the need to follow the country’s IPC guidelines, which include a training for trainer component. It was noted that all health facilities provided Continuous Medical Education (CME) to HCWs; and this training focused on topics agreed upon following assessments conducted within departments or the entire health facility. Despite its regularity and familiarity to all, the CME course did not primarily focus on COVID-19 IPC.

*“There was no corona, but when corona came, we had to twist it [the CME] again. We had to put on some other programs which were like freshen and put what? CORONA inside. Because people [HCWs] had to know more about corona very fast”,* Participant 7, IPC focal person.

#### High workload

The services provided by HCWs were consistently in high demand, often surpassing their capacity, leading to long lines of patients awaiting attention. Many HCWs worked tirelessly without taking breaks throughout the day, and for some, shifts were of short duration. Consequently, some HCWs found themselves skipping recommended IPC procedures in order to attend to as many patients as possible within the limited time available.

*“By that time [of the pic of the pandemic] the workload was like [high]. In my hospital still we have many patients and the staff is few; the staff who serve all those people. So, you find that they [HCWs] were overworking”,* Participant 13, IPC focal person.

“*You need to work on the other [the patient] so this time of going to wash hands can save someone’s life*. *So*, *sometimes you may not go*, *you just need gloves and you don’t wash the hands and save someone’s life”*, Participant 1, Lab technician

#### Lack of resources to buy PPE

HCWs perceived purchasing PPE as a significant challenge. For instance, out-of-pocket expenses to buy masks was not considered a favorable option.

*“At our facility here we buy masks for ourselves; in most cases we buy masks. We even buy gloves. Umm… I can say most of the PPEs we buy. Umm… I remember once in a while the ministry helps us with some few; once in a while”,* Participant 2, Lab technician.

According to participants, HCWs preferred either not to wear a mask or to use it for multiple days, even when it became dirty. They also mentioned other contributing factors, such as feeling safer due to vaccination, the decrease in the number of COVID-19 patients, discomfort with certain types of masks, difficulty breathing, or the challenge of communicating effectively with patients while wearing masks like N95 respirators. These factors, combined with the idea of having to purchase masks, contributed to resistance toward wearing them.

*“We use them, we reuse them very many times. Like you buy like on a Monday, I can buy three masks and that is for a full week. Yes, you buy, you use like Monday to Tuesday or Monday to Wednesday, if you are not safe a lot you put it on again”,* Participant 8, Nurse in charge.

Gloves were occasionally reused when attending to different patients due to scarcity. In the interest of self-protection, some HCWs ended up reusing gloves without realizing that this could serve as another route for transmitting infections from one patient to another.

*“When there are many [patients] it is not easy to change you may even forget. Because you are looking at [the] time, you have a patient in bad condition you forget and you feel like let me just not put on these gloves. But again, by doing that you may also be risking. But most people [HCWs] forget and people are not used to that [putting on gloves]”,* Participant 2, Lab technician.

#### Being vaccinated against COVID-19

The high COVID-19 vaccine coverage among HCWs influenced their attitude, leading to reluctance in compliance with other preventive measures.

*“They are not behaving the same way, with [the] coming of vaccine, of course, most of the people [HCWs] got vaccinated. So, based on their knowledge, of course, for them their knowledge tells them that when you get vaccinated the risk of COVID reduces”,* Participant 11, Nurse in charge.

Although it was well emphasized that vaccination reduced the risk of severe forms of COVID-19, many HCWs viewed this as sufficient to guarantee their safety. It is important to note that HCWs were already aware of the benefits of vaccination in general and understood the protection provided by COVID-19 vaccines. However, the determination to adhere strictly to recommended measures was not fully embraced. Meanwhile, despite the evident benefits of vaccination, in some health facilities, ongoing efforts to raise awareness among HCWs persisted, with the aim of ensuring full participation from all staff members.

*“People [HCWs] now have that mindset that okay umm… since I have got the vaccine umm… I am safe because with that, when you get the vaccine even if you get COVID you don’t die, you don’t suffer severe condition, so it has changed people’s minds,* Participant 2, Lab technician.

#### Inaccessibility of hand washing facilities

Many HCWs noted the challenge of constantly moving back and forth to the sink to wash their hands while attending to patients. Some handwashing stations were not within reachable distance, prompting them to prefer sanitizing their hands instead.

*“I feel like sanitizing is easier just sanitize [….], but now as far as I have a sink outside it requires me to first step out getting to a sink”,* Participant 14, Nurse in charge.

*“Sanitizing you take a short time but when washing hands*, *you have to wash very well*. *Yes*, *especially when they [HCWs] are busy*, *when they have a lot of patients [washing hands is not easy]”*, Participant 5, Lab technician.

However, participants highlighted that the swift rise in interest toward hand rub sanitizing marked a significant achievement for hand hygiene campaigns during the COVID-19 pandemic, especially considering the existing challenges with hand washing in their health facilities.

*“They believe that using sanitizer, using something of 98 percent antibacterial kills corona than water which you are not even sure of”,* Participant 7, Lab technician.

## Discussion

This study explored barriers and facilitators of IPC compliance among HCWs in Kampala City. IPC, as core component of quality care, gained significant attention during the COVID-19 pandemic, particularly for ensuring HCWs’ safety in hospitals. Participants in this study reported that during the pandemic, facilitators of IPC compliance included the fear of contracting COVID-19, social pressure, and the moral obligation felt by HCWs and the provision of enough IPC supportive equipment by the health facility management. Compliance with IPC varied depending on the evolution of the COVID-19 epidemic, with HCWs exhibiting greater compliance during the peak of the pandemic, when more cases were registered in their surroundings. Among the barriers to IPC compliance were inadequate knowledge of IPC among HCWs, high workload, lack of resources to procure PPE, vaccination status against COVID-19, and, lastly, inaccessible handwashing facilities. The findings of the study suggests that there is significant room for improvement in the implementation of IPC measures by healthcare workers in health facilities.

Key informants reported that HCWs did not want to experience the guilt for not following the set guidelines during the early stage of the pandemic, which compelled them to use masks. They were motivated not to ignore IPC regulations because they believed it would motivate the community to follow IPC recommendations intended for the general public. Similar sentiments were expressed by participants in a study conducted in China. Yang, Wang [4] which reported that the level of compliance with mask usage was influenced by how HCWs wished to be perceived within the community. This attitude suggests that, in addition to self-protection, HCWs wanted to appear knowledgeable and make a good impression on patients. As it has been stated, HCWs adhere to all required IPC procedures to ensure patient safety, particularly when they are aware of being observed by others or the patient [29]. Making sure that patients have enough knowledge on IPC can, therefore, serve as a model to stimulate HCWs in the health facilities to be more compliant.

Participants emphasized that the use of gloves and other PPE should be considered as a self-protection measure, as highlighted by HCWs elsewhere [29,30]. However, there instances where one pair of gloves was used on multiple patients. As stated by study participants, the intention of the HCW was not to harm subsequent patients, but rather resulted from a lack of sufficient PPE or information on the cost implications of healthcare-associated infections, including the spread of COVID-19. Further analysis suggests that the lack of knowledge and information on the use of PPE could be a result of poor dissemination of guidelines and standard operating procedures to HCWs. This was noted since the beginning of the COVID-19 pandemic and even exist during normal times when guidelines are frequently modified but not effectively distributed to end-users [29,31–33]. As reported in our findings, the consequence of this situation is poor compliance with IPC, leading to an increased risk of infection and the potential spread of infections within the health facility and beyond. Therefore, more effort is needed from health systems to ensure that HCWs received refresher training, and most importantly, have access to updated guidelines [33,34].

If not due to scarcity or laxity, participants mentioned that the improper use of gloves may be justified by the workload in health facilities. For instance, some lab technicians reported the belief that gloves alone provide sufficient protection, thus negating the need for handwashing. This perception may result into glove usage among laboratory personnel than other HCWs [35,36]. However, in other areas of the hospital, different studies suggest that workload can hinder proper glove use and reduce IPC compliance during emergencies [37–39]; leading HCWs to skip certain IPC steps and prioritize saving lives. Therefore, there is a critical need to focus on the patient-to-HCW ratio in healthcare facilities and enforce IPC measures accordingly.

Participants reported that the lack of knowledge on IPC was linked to inadequate training and mentorship, which are known factors contributing to poor IPC compliance among HCWs [6,19,31]. Previous studies in Guinea and the Democratic Republic of Congo noted that having trained staff on IPC was associated with better performance during outbreaks [17,40]. Meanwhile, aside from glove usage, which is also a standard precaution for HCWs during the COVID-19 pandemic, mask usage and hand hygiene were widely promoted in the community and healthcare facilities. Wearing a mask consistently or practicing hand hygiene did not necessarily require training or special communication during the pandemic, but rather continuous monitoring or simple reminders. Justifying inadequate compliance with IPC measures solely by the lack of training may not be appropriate unless participants referred to the use of all PPE, including a full set of protective gear used by HCWs in COVID-19 isolation units. The positive change reported by participants regarding IPC best practices will, therefore, require more effort from the health system, healthcare facilities, and HCWs for sustainability. However, this positive change is challenged by various other factors [26].

Participants in the study advocated for more training, expressing the need for a well-established training scheme and a fully functional IPC program during outbreaks and normal times. This has been documented in the past as an important factor for IPC compliance [35]. Similar recommendations emerged from the devastating Ebola outbreak in West Africa in the previous decade [16]. In our context, a well-functional IPC system would provide valuable insights to the community of HCWs who rely on various sources of information to guide their use of Personal Protective Equipment.

From the study findings, participants reported that HCWs believed that the COVID-19 vaccine provides sufficient protection, which was viewed positively as it likely increased vaccine acceptance among them [41]. The prioritization of HCW vaccination was highly sought after due to their high exposure while providing care to COVID-19 patients [42]. However, participants also noted that HCWs’ compliance with IPC measures had decreased despite knowing that the vaccine reduces the risk of severe infection but not the risk of reinfection or transmission. This attitude was considered inappropriate, considering HCWs’ central role in the community and their frequent contact with individuals visiting the hospital. Nonetheless, their high level of vaccine acceptance underscored their reliance on vaccination benefits and a positive attitude toward new scientific research findings.

This study had some limitations, including the missed opportunity to gather firsthand experiences from healthcare workers implementing IPC practices, which would have allowed for triangulation of the collected information. Secondly, designing the study and presenting the results and discussion using an established model, such as the COM-B model, would have facilitated comparison with other studies and provided a clearer framework for guiding interventions. However, a strength noted in this study is that it was conducted during a time when health facilities were at high risk of receiving COVID-19 patients, providing valuable insights based on recent experiences. Participants were also able to provide a holistic view of the situations in their respective health facilities.

### Conclusion

This study found that during the COVID-19 pandemic, the barriers and facilitators of IPC compliance in health facilities in Kampala City, were largely in line with the perceived risk of being infected by the virus. The support provided to HCWs was highlighted primarily involving the provision of IPC supplies and adequate training on the importance and proper use of PPE. This indicates that addressing the spread of COVID-19 among high-risk groups such as HCWs necessitated a comprehensive approach to IPC in health facilities. Equally important, HCWs, the hospital management and the government should coordinate their efforts to ensure IPC compliance during the COVID-19 and other pandemics.

## Supporting information

S1 Data Transcripts for the 14 interviews. (PDF)
