# Supplementary material for: Barriers and facilitators of compliance with infection prevention and control measures during the COVID-19 pandemic in health facilities in Kampala city, Uganda
Source: PLOS Glob Public Health. 2024 Dec 9;4(12):e0004021. doi: 10.1371/journal.pgph.0004021 (PMC11627369; doi:10.1371/journal.pgph.0004021)
Supplement: S1 Data — (PDF) [file pgph.0004021.s001.pdf]

TRANSCRIPT FHC

INTERVIEWER:

Introduction: thank you so much for allowing to attend this interview with us. Today is the 4th of April 2022 and we are at …… umm like I have said the interview is going to be recorded. I would like to ask you few questions about how health workers behavior in the facility.

Moderator: Umm what are the reasons that could hinder proper use of PPEs among health workers generally in the facility?

Respondent: the scarcity umm most important scarcity because you will try to use you will try to minimize should I say wastage something like that umm someone will not use the masks well. it is supposed to be double masking you will use one mask because what you might save from the other person.

Probe: and what are some of the barriers or challenges that would hinder health worker from wearing their mask or practicing handwashing hygiene?

Respondent: I don't think there's a place with no water unless when there's no water availability. The way the hand wash institution is we've seen people have to touch and then they have to wash hands. So that's why we opt for sanitizer instead of washing so the way the washing station is also far.

Probe: So are you the health workers around able to access PPEs?

Respondent: yeah for us we can.

Moderator: What are some of the PPEs that are accessible?

Respondent: We have masks, we have many hand washing places and manipulated umm the disinfectant. Sanitizer are all over the place and you will get mask; those days we used to get face shields but because of reduction in number of cases so that was stopped. We used to have umm

the coats that we put on all of us to avoid infection to help in infection control in most cases but because of there's a trend in the pandemic so they opted to leave some.

Probe: And what are the available masks

Respondent: umm We used to have N95, for us we used the disposable ones and then K95 was given to everyone and then the disposable ones, the lab guys used the N95.

Probe: Okay what the other things that they used?

Respondent: umm the lab guys

Moderator: yes

Respondent: They used the lab coats; they have gowns those gowns, masks and facial shield.

Moderator: Are there times when you were not able to access the PPEs?

Respondent: umm of course it possible (laughter's)

Moderator: umm what about in the beginning during the severity of the pandemic in the first wave. Was the facility able to access the PPEs?

Respondent: It was actually. You could not see the patient without PPEs even if a client come we have mask. There was zero tolerance with the PPEs.

Moderator: And did the severity of the pandemic in any way affect the facility in terms of PPEs use among health workers?

Respondent: Not really because we did trainings and in the very first wave we had a training vigorous all the health workers in the facility to help them IPC, the different measure to take we got them from …, KCCA to help out

Probe: Okay, how about the vaccination campaign has it in any way affect IPC compliance?

Respondent: I will say so, many have been vaccinated and the pandemic is now in its way out so people are now relaxed.

Moderator: okay how about in the fact that some clients come in with symptoms, does it in any way affect IPC compliance?

Respondent: the clients are screened because they screen the temperature, we put pressure whether you don't have a mask, the person at the gate will make sure you have a mask and the person at the reception will make sure that you sanitize.

Moderator: Would you say they need to protect others, especially the clients coming in the way they protect themselves? That prompts them to use the different PPEs?

Respondent: Anyway, it is not even the need to protect others but to protect themselves because during the first wave we had around 8 people but in the second wave had like 14 so people, and now they are minding about themselves.

Moderator: okay so we go to the second theme and I would like to ask you few questions about the different factors that motivate health workers to comply with IPC. Umm what are some of the reasons that you have seen yourself that motivates health workers to use the PPE?

Respondent: what are some of the?

Probe: reasons that you have seen that motivate health worker or yourself ….that motivate people to put on mask, to disinfect, to wash hands

Respondent: umm think infection control is paramount even if there wasn't covid I mean its paramount even if there was no covid and there was sanitizer around. But we were not used to mask but for sanitizing and hand washing it was paramount because (background noise) a lot…

Moderator: okay apart from IPC being important and paramount and a requirement for practicing IPC, now that we have been using masks, what are the other reasons that motivate people or health workers to put on and hand wash

Respondent: me I have one reason, it's more of prevention control. I have worked in ICU, I have worked in theatre and different umm like okay it means is not that well I always try to avoid like coming in contact with infection as much as possible it's more of self-protection.

Moderator: okay umm do you support health workers to comply with IPC?

Respondent: yeah we do

Moderator: what kind of support?

Respondent: trainings and mostly trainings. The other support is giving the PPEs and soap

Probe: how often do these trainings occur?

Respondent: at least once a week every Friday.

Moderator: okay what are some of the IPC approaches that used by management to make sure that staff apply the different maybe supplies that are in place to help practice

Respondent: Of course by the deputy director is spot checks sometime

Moderator: is there any support that you are given to the health workers when they are going back home?

Respondent: am not sure, okay anyone who gets covid, we ensure that they are followed up and know their plan for their family members

Moderator: are the different water stations accessible to everyone even with people with disability?

Respondent: I would say accessibility is low because it's a step out….. you know you just have to step out so let me say, I don't think it favor people with accessibility.

Moderator: so during this pandemic period, have there been preferences for health workers between having to use sanitizers and then having to hand wash

Respondent: definitely

Probe: what's the preference?

Respondent: umm personally I prefer sanitizing more than hand washing, because if I know I have disinfectant I have everything I want to use.

Probe: so for the other health workers what would be the choice?

Respondent: but most of them sanitize, but more than hand washing. I do hand washing when am sure am going to spend the next fifteen minutes when I wash hands not fifteen okay close to five to washing hands. I don't want to wet hands and then

Moderator: okay so do the different heath workers have access to the sanitizer?

Respondent: yes everyone has in there

Moderator: okay let's go to another theme which is about PPE stock out. And are there days when the health facility experienced the shortages of PPEs?

Respondent: at least never I have not seen any.

Probe: Before the outbreak or during the out break

Respondent: have not seen any stock out unless we try to do stock takes if we realize we are running out stock out.

Moderator: are there days when they had to reuse particular mask

Respondent: (laughter's) because we are given at least two pairs like a pair of masks every day the KN95 and the disposable one everyday

Probe: so there low chances that they could even buy for themselves

Respondent: because they have to give you for taking home and in coming back the mask your going to use

Moderator: so that is for themselves not family members?

Respondent: not really they are supposed to care for themselves

Moderator: are there days when health workers need to practice hand washing due to sanitizer at the different duty stations

Respondent: yes (background noise)

Probe: why? who did this?

Respondent: someone when sanitizer was used

Moderator: what are your recommendations for the health workers in the facility or to policy makers in regard to the implementation and

Respondent: umm personally I believe that first of all it should be in private you know when it's no for Government or what but for people around everybody must be vaccinated. Let them put in

more feeling for people to understand why they should be vaccinated not just pushing because human beings are never animals

Moderator: is there anything else that you would like to share with us? Any other itching issue that maybe you've seen or noticed?

Respondent: umm I just want to say IPC regulation is not there end everything is for improvising so I think facilitating IPC (background noise). We don't prioritize certain thing we believe there not there yet it's what are these thing infection is coming from. So I think we are not having priorities that we dwell more on treatment….. I believe in prevention.

Moderator: so thank you for sharing your experience with us

TRANSCRIPT: SSMD

INTERVIEWER:

Introduction: so thank you very much for allowing to attend this interview with us. Today is the 26th April 2002 and we are in Luzira. Umm we have a few questions about IPC and the interview is divided into four themes so we shall be going through each theme at a time. So the first theme is about the barriers associated for PPE and hand hygiene. So am going to I will be asking you questions about different barriers that the health workers in the facility face ever since the pandemic started.

Moderator: So the first question is what are the reasons that you think could hinder a health worker using PPEs?

Respondent: Ignorance about the PPE. Other health workers don't know like how to use them, what is needed for what purpose yeah it is one of the things or the reason, yeah

Probe: umm how about if they are sensitized. Have they been sensitized about the use of PPE to know that now that we are in covid pandemic and we have how infectious they should be able to use the PPE

Respondent: umm some of them have been sensitized but some of them have not because some have gone to those courses about the use of PPE but others have not got a chance going to attend but in case they are given a chance to I think it will improve the use.

Moderator: and what are some of the reasons that you think that hinder the same health workers from umm proper hand hygiene given the fact that we are in the covid period

Respondent: I think I have not got you well

Probe: I mean… what are some of the reasons that can hinder proper hand hygiene like washing your hands maybe with soap and water or with sanitizer?

Respondent: umm maybe like for example if the sanitizer or the soap are not provided can also hinder, yes. Some of the health workers its their careless they don't care they don't care.

Then others when they are busy or when they are tired they can forget yeah so that is what I think.

Probe: but have you got complaints were like the other health worker are complaining that so and so doesn't wash hands, doesn't use sanitizer

Respondent: yes I have heard like two health workers

Moderator: umm are you able to access all the required PPE whenever you needed or whenever the other health workers needed like the gloves, the masks

Respondent: yeah most of the times we access them umm the only problem we find like when the store manager is gone and some of them like the gloves are done that's where the challenge comes in but when he or she is still around of course we have to go there they are accessible.

Moderator: and in the event that they are not accessible (background noise) what do you do?

Respondent: umm we improvise yeah we improvise like we have alcohol swabs so sanitizing we use alcohol swabs.

Moderator: okay are there chances where a health worker had to buy for themselves during that period of covid

Respondent: it was like once when we didn't have and we bought from out, yes.

Moderator: umm what did about the severity of covid-19, we all know covid-19 has been severe or is severe umm are there chances where or cases where umm someone knowing that covid-19 is here and it has affected the way they thee compliant in terms of implementing IPC?

Respondent: umm come again

Probe: for example am saying umm knowing that covid-19 is severe, okay has it in any way affected IPC compliance either in a good way or bad way?

Respondent: yeah some of the health workers who were careless have tried to improve actually most of them now have been influenced umm infection control has improved, yeah

Probe: how about now that we are in the era of vaccination, has it in any way affected IPC compliance

Respondent: yeah it has affected it because some of the health workers no longer put on masks, no longer take issues serious like the other time when covid was severe. Yeah they have relaxed a bit

Probe: why?

Respondent: I think vaccination you vaccinated and you get covid or get infected with covid you don't become badly off or it doesn't affect so much your vaccinated

Probe: Alright, how about the need to protect others. Does it in any way influence a health worker to say let me wash my hands, let me use sanitizer, let me put on the mask

Respondent: yeah it does to some yeah but most of them are no longer serious like the other time, yeah

Probe: could it also be because in the covid and here for long

Respondent: at some extent they are now used because others say they now have immunity against covid.

Moderator: umm we go to another theme which is about motivators of IPC. What are some of the reasons that you have seen so far that motivate or influence the health worker to comply to the different IPC measures

Respondent: its because of the severity states that patients have gone into yeah. They have seen patients dying, they have seen patients suffer they have seen so many things so that has been also the motivator in them because of the fear.

Probe: how about among them as health workers

Respondent: among them!! Umm what I can say maybe there's one who got covid yeah and they feared eh because most of them didn't but one who got umm stayed at home for some time it affected her eh.. so that's what I can say but most of them are in yeah

Probe: how about in terms of availability of the PPE? Does that affect them in any way? Like knowing, that the hand washing station are there umm the soap and water are their, how has that umm motivate them to be compliant?

Respondent: there have motivated them because at least whenever they need them it has increased than the other time. When covid was not there because before some health workers used not to put on gloves when handling patients but at least now you rarely a health worker not putting on what gloves ehh at least they have improved that.

Probe: you also mentioned that …..does audit, how does that motivate the workers to be IPC compliant

Respondent: it motivates them (background noise) in which way like positively

Probe: you know it (laughter's)

Respondent: it has motivated them because for example ….. there's the money it gives PHC funds so through that at least we have tried to buy more PPEs than before yeah. That's good because whenever they want, there always available compared to the other time

Moderator: you mentioned that there some health workers that have not ben trained or they are ignorant about IPC in general. So has the health facility support to them

Respondent: yeah they have tried because those who go for training they set a day and they come they teach others yeah but because you know teaching your fellow health worker is not easy than a person who has come from out but at least they try to come and teach others.

Moderator: what are some of the approaches the IPC approaches that management uses to or apply up to date to make sure that you know your IPC measures are in check in other IPC guideline

Respondent: umm support supervision eh they know they provide like …… and other Organization to come and inspect and help where necessary then provide like with the bin liners thins yeah

Probe: what is …….?

Respondent: ………..

Moderator: okay thank you very much. Is there any support that is given to the family members of the health workers like when they go back home let me say that at the end of the month at the end of the week, family support like sanitizer take to your family

Respondent: not really

Moderator: umm are hand washing station and sanitizers accessible to all health workers in the facility?

Respondent: yeah because every department has a center

Moderator: is there difference among health workers between sanitizer and hand washing?

Respondent: like a difference?

Probe: yes like choosing between hand washing and sanitizing

Respondent: actually (laughter's) most of them choose sanitizing than hand washing but because of time taken when hand washing. When sanitizing you take a short time but when hand washing you have to wash very well, yes especially when there busy when they have a lot of patients.

Moderator: umm but what is generally recommended?

Respondent: hand washing

Probe: so do you think we need maybe to sensitize them more on hand washing?

Respondent: yeah (laughter's)

Moderator: do the health workers have access to umm the SOPs of IPC on covid-19 PPE use in there different work station

Respondent: yeah they do

Moderator: okay go to another theme which is about PPE stock out and would like to know are there days when you have stock outs or shortages of PPE in your health facility

Respondent: yes like what I told you before when the store manager is not around the health workers have not ordered in time yeah but they are always available

Probe: you mentioned that when that happens, you improvise, what are the other measures that you would adapt or put in place to make sure that you know what even if the store manager is not around we can still function?

Respondent: function umm for example you work on a patient let me give an example when your putting a cannula, you put a cannula but immediately you wash and sanitize, yes

Probe: is this practiced or is this implemented by all the health workers?

Respondent: umm some of them umm seems in most cases the PPE are available so it is some times and some of them not all

Probe: are there cases where a health worker has to reuse a mask or a glove?

Respondent: a mask a glove is used one time but mask (laughter's) umm they see every day they provide a new one because we use for a week.

Probe: you have not had cases where a health worker has had to buy for themselves glove or PPE or masks

Respondent: they have (laughter's) a mask they have because some of them if it becomes dirty even before a day they change ehh because they use at least a day per day one or a week they give us seven yes so its up to you but in most cases they give us.

Moderator: okay you mentioned that there's preference of umm sanitizing and hand washing, are there days when a health worker umm may not sanitize due to lack of a sanitizer in the health facility

Respondent: not really it is always available

Probe: you've not had cases where umm sanitizer is not available

Respondent: um um it's available

Moderator: what are the some of the recommendations that you give to health workers to the policy makers regarding IPC implementation or the guidelines or the SOP

Respondent: even the policy makers?

Moderator: yes

Respondent: sensitization at least they make time

Then trainings at least every year they should be training actually like twice because other health workers get a chance to train others don't, yeah

Then health workers they should be experience because at least most of them know but they do it stubbornly and I um. I will do I will not do this and yet they know the advantage and disadvantages just we need to improve, yeah

Moderator: umm one last thing now that you made that recommendation you said the PPE they come to do audit how often does audit is carried out?

Respondent: every year.

Probe: do they share the feedback with you about your performance?

Respondent: yeah they do. They tell the administrator then others come directly like yesterday I had one he came here and recommended and he left me then I shared to the administrator too.

Moderator: well is there anything else that you would like to share with us?

Respondent: share with you? I say thank you for coming in case have any other materials to provide you can provide, eh in case you have any material to share with us please come and share.

Moderator: thank you so much for your time we really appreciate the information you shared with us.

……..

TRANSCRIPT: SL

Interviewer:

Good afternoon once again

P: Good afternoon.

Thank you for being part of this interview. We are discussing about factors associated with compliance with infection prevention and control measures among healthcare workers, to minimize the risk of COVID-19, in Nakawa division, Kampala, Uganda.

I will go straight to the first question.

What the some of the factors that would hinder or block healthcare workers to use gloves and masks during this COVID-19 time.

P: that would block them for using gloves?

I: Yes, for gloves and masks

P: They are using; healthcare workers in my hospital they are using them. Especially during this pandemic. In order to prevent those pandemic, in our facility they are supposed to use.

I: What about hand hygiene, are they also washing hand ?

P: It is already here at the health center. And it is not only for the healthcare workers, everyone who enters the health center is supposed to wash their hands.

I: Don’t you have some people who do not comply with that?

P: No, because it is their responsibility. First for the health workers, they should know that it is theirs. First, they should know that COVID has come and washing hands is for their hygiene and they should teach other also. And even if COVID is here or not those things have to be there. Those are the fundamental procedures they are supposed to follow.

I:Are you able to access the been supply like masks and gloves?

Since then, our facility has not been provided with any support in terms of primary health care or any other funding's. So at this facility, the management has decided to provide because it I necessary.

I: Do you think that the COVID-19 vaccination has changed healthcare workers behavior ?

P: A far as I am in Uganda, I have not seen any changes that they vaccine will help to prevent the infection. But at first they were not willing to take the vaccine. But after I had come, because I am a new manager, and I have come like two months before so I have instructed my staff to take the vaccine and I think 99 % of them have taken the second dose.

I: Do you think that since they vaccinated they are more reluctant in using masks or gloves?

P: Ummmh, yes; some of them are reluctant they think that since the vaccination is finished the immunity power has built up in their body. Yes some relaxation is there but we advice them on a daily basis to use…that is why I am coming back to the same point, we don't look only at COVID-19 but other infections as well.

I: Okay, what about the belief that COVID-19 is not severe? Do you have people who have that belief?

P: I am not sure about the belief, because you can see for example two months back the cases had gone up again they went down. But now like last month and now we are not hearing much about symptomatic cases, because we use to treat normally up to December and middle of It we were doing the testing, primary testing. But in the last two months we are not getting any cases like that. The children like of 5 years and below are coming with symptoms like fever cough flue, and I though we are maybe the one having them but I mad an analysis and every health setting they are getting even the schools, children are having those symptoms. I think it is like kind of influencer. Some people are making some research, but those are the messages we used to hear.

I: What about the belief of healthcare workers?

P: The healthcare workers, they do not follow, they just carry their own routine.

I: Do you think that some healthcare workers may comply with IPC measure because they need to protect others?

P: Because they have to comply, they have to follow what is on IEC material and all that. The COVID-19 we have now is not the one we had in 2019, it keeps mutating mutating , and we are in 2022. Another may be mutating, and it may come and we don't know.

I: Do you provide any kind of support to health workers about wearing masks and hand hygiene?

P: We used to provide masks, on a daily basis, and they hand washing procedure, water and soap is everywhere. Gloves . these are the basic things. But full IPC, those materials, are not available, it is very expense to buy. And now, people are reluctant, I don't think that we can buy it and they use it. But now these basic things, mask, gloves, hand washing those we provide.

I:Between hand washing and sanitizing, do you think people in your facility have any preference?

P: we stress on hand washing. Because sanitizers are different types. And the fundamental procedure is and washing. Every medical procedure health works do, they are supposed to wash.

I:Do you have access to COVID-19 standard operating procedures?

P: I personally as a manager I have a soft copies, elsewhere we have IEC materials.

I: and what is your approach now when you want to teach health workers about IPC ?

P: As a manager, I tell my staff on a daily basis to follow the measures, to try to remember the disease there and here and it can come back. We need to have that awareness.

I:Are there days you had experienced the shortage of PPE?

P: We have not been provided by the Division, as the management we make sure that we purchase everything. It was not out of stock.

I: My last question is about your recommendation, to manager, healthcare workers in other facilities and the government

P: My recommendation first of all. It is for the health workers, they are not like other people, they should follow what they learned at school. They are suppose to put it in practice. Many pandemic will come many pandemic will go, they different everything because life is important we need to understand the value of the life, and we need to carry the health care services in a good way.

Because every patient is of value to us, we should not follow that thing of money, money will come and money will go, we need to treat patients with value.

For the government, I think the county depends on donations, they need to utilize that money properly. For the healthcare system, the ministry should utilize resources properly.

I: Thank you so much for sharing

P: thank you too, personally I happy for having this chat with you.

……

TRANSCRIPT: ROK

INTERVIEWER:

INTRODUCTION: thank you for being part of this interview, today is 14th April. It is11:38. We are in Kinawakataka. Thank you once again we are discussing about factors associated with compliance infection prevention and control measures among health care workers to minimize the risk of Covid-19. This interview has three themes; the first one is I shall ask about barriers for use of PPE and use of handwa…, hand wash, umm hygiene, the second one will ask about the motivators of IPC compliance and the third one will focus on PPE stock out in case you faced the challenge. So directly let me ask the first question which is about barriers for use of PPE and hand hygiene.

Moderator: what are some of the reasons that hinder proper use of mask and gloves among healthcare workers in your facility during this Covid pandemic?

Respondent: Actually you know most healthcare workers here they were not used to putting on gloves and they were also not used to putting on masks. So when corona came, and started enforcing the guidelines from the ministry of health it was a problem in that someone put on gloves just within one minute the person has removed. Someone put on mask like someone can give the person a mask like **N95** to put on even someone brings it back, umm this one I cannot breath you give me the lighter one so it was like there just getting used to what ….they just getting used to mask, these new measures of what of Covid.

Moderator: okay so if somebody report that they don't feel comfortable using the mask

Respondent: using the mask they even brought them back.

Probe: okay any other reason maybe you've seen so far apart from breathing

Respondent: apart from breathing maybe also there some others um who have say who talk in about the mask so when these people goes outside there from here, they also of course come with the same thinking that what we are giving them is not the right thing so best thing is for them to go just the way they are.

Probe: okay, okay

Respondent: these people have their own doctors in the community that they listen to very well. So that in such a way that if you don't explain to them uh what the ministry wants they won't do. They end up being taken up by those people… those main people in the community who are strong voices, yes.

Moderator: but I was expecting that healthcare workers you are always in contact with your fellow healthcare workers you may give them more knowledge, teach them what you know from the science than what is given from the community

Respondent: yes. At the beginning it was not easy because those people's voice it was too much and they are even from the ministry of health. It was like that initially because you know truth always takes time to come out uh? It was all about time that we keep on telling them telling them and they kept on seeing practically what is on the ground that's when they started accepting our words and they follow us.

Moderator: umm I would now shift to hand washing and hygiene use of sanitizer or hand washing soap. Umm what are some of the barriers that you have faced so far?

Respondent: so far, we have faced the issue of soap more softly because at times you can put soap there at the point there someone then after some after like a client vising you, if the person washing hand can easily go with it. Because soaps are always small like this and it went repeatedly until

when we started what we developed the idea of using liquid soap in a tin that you tie there uh that is when what that is when we now stopped people from taking it.

Then another thing also the shortages. Shortages of soap was there because it was in high demand by that time.

Probe: high demand

Respondent: yes. Then also another thing, since we are using only one point per washing entry for general people sitting down there even coming inside here uh that thing could get spoilt very first but again you have to take some time to repair it. Eh. So those are the things.

Moderator: but generally healthcare workers did they report to you some challenges there facing or there you saw them about them washing hands

Respondent: ehh they were also not used to using what to using liquid soap that all they could report to me when soap is used uh uh musawo you bring for us soap not liquid soap.

Probe: uh they want the real soap

Respondent: they want the real soap because soap has that kind of taste in the hand after the hand dries, he has to apply something else

Probe: okay okay it has kind of smell

Respondent: not a smell but the way it can make the hand dry eh so that’s what they were facing

Probe: ehhh they see that the hand is very dry?

Respondent: dry and they have they want to give to put some Vaseline on it again. So that’s what they were facing

Moderator: is it when they wash hands many times or just one time when they feel that the hands have dried.

Respondent: in fact initially it was once but after that one we realized that we need to dilute it for more but still even after diluting you see some washing like six times that’s in goes and comes back

Moderator: now are you able to access PPEs, masks and gloves when you're at the facility during this Covid pandemic

Respondent: yes

Probe: you had access?

Respondent: I have access constantly

Probe: even that problem of hand washing

Respondent: I did not face the problem of mask but I faced the problem of gloves. By that time actually even first of all even without corona they brought us in the country so the price should have even they were not available in the market so coping corona it was not easy to get what to get even gloves this side.

Moderator: I think I will come back to that later but I want now to here from your point of view, umm do you think that healthcare workers take Covid -19 seriously so that they comply with whatever advice you give them?

Respondent: In my view, uh! It is actually individual. There some healthcare workers which takes Covid seriously only when they hear that today they have brought one thousand in twenty hours, two thousand in just twenty four hours but when just a slight but when like it is just a slight day, when the numbers like they get tripled just like yesterday they were just one thousand in just like twenty four hours and then they get seven hundred tomorrow they get four hundred even they are seriousness; corona also reduces and they become relaxed.

Moderator: I understand. There also following the trend?

Respondent: they are also following the trend. When it is high they become very serious when it is low there not that serious. I think you can even see today and if not all, I think you have seen only me with mask (laughter's) that is how there.

Moderator: okay. When they say that it is serious then they. Now I have got also a feedback about umm one healthcare worker who was saying that in his community once they get they got the new case there, everyone became serious. Is it the same feedback you're having?

Respondent: Yes. You know the first case of corona, they went on but the second phase they call it what?

Probe: second wave

Respondent: when the second wave, came it got them the way I told you… they were all relaxed unfortunately they got eighteen people at ago um? So that is when they became very very serious when they heard that eh! This one has gotten I think am next that's when they became very serious.

Moderator: okay (laughter's). Umm what about the vaccination. Do you think that it has influenced the way people take Covid-19? How they wear mask,

Respondent: the vaccination?

Probe: yes how did the vaccine and how people wear mask.

Respondent: ummmmm as staffs or everyone

Probe: the staff

Respondent: as staff. It has not changed that much because we had already told them we had already told all of them that not just you're doing vaccination, your taking vaccination it is not supposed to stop you from wearing mask because vaccine only reduces the chance of you going to ICU or getting admitted but does not stop you from getting corona so even if they go and told and got the vaccination still they come back wear their mask knowing that at the back of their mind that it is there it can easily get me so if that the approach I should not do what? I should not get it

Moderator: okay. Umm I appreciate that too but now what about the need to protect others. Umm you may know that as a healthcare workers you're the center of the community. Everyone comes to you so did can that be a motivation to make you do the right thing so that you don't spread the infection to everyone?

Respondent: yeah it can be actually motivation knowing that before the people have entrusted you to be there…. What…. to be their healthcare worker. You must do the right thing so that they see you. Musawo always do that thing why can't I copy and do the right thing so you an be like so it's just like that's a good motivation.

Probe: but is there anything you do to motivate your healthcare workers?

Respondent: to motivate them like?

Probe: to put on mask, wear gloves, umm. What are some of the things you do to motivate them?

Respondent: actually the only thing I do to motivate them is not giving them money or what, I just make sure that I am doing it rightly so that they see on me like every time I come I put on my mask even if Covid is not there little or high they always see me on the mask and in the beginning they could complain like eh! Musawo every time you're on mask every time mask but after the second wave they appreciated that let us all be on mask so acting in the right way helped me to motivate them and also do the way what the way we want.

Moderator: okay. Ummm what about do you provide trainings?

Respondent: trainings, yes CMEs

Probe: CMEs. Okay. Umm are they there all at the time or

Respondent: it is twice a month

Probe: you do them twice a month? How are they organized?

Respondent: like it depends we can initially when there was no corona but when corona came we had to twist it again we had to put on some other program which were like the fresher's and put what corona inside because people heard to know more about corona very first.eh so we put a schedule like the whole year, uh! Just know that this one today there going to be what malaria, next week is going to be hypertension then the next four months it's going to be diabetes like that but when corona came, we had to shift others aside and put corona.

Probe: eh they were focusing on corona?

Respondent: yes

Moderator: now do you have access to water all the time?

Respondent: access? Water is always there.

Probe: so can you use that easy enough to motivate your healthcare workers?

Respondent: yeah it can be because the fact that there's no water, the fact that there's water, I don't think there's any excuse that ah ah excuse of not washing hand.

Moderator: umm now do you find that there's any difference among healthcare workers between sanitizing and hand washing? Is there anything they prefer?

Respondent: yes yes yes. They prefer sanitizer than hand washing

Probe: why do you think so?

Respondent: because most of the time the it's just like they believe that using sanitizer using something of 98 percent antibacterial to kill corona than water which you're not even sure of.

Probe: okay they think that sanitizing is

Respondent: sanitizing is better as you hear the name, sanitizing than washing hands.

Probe: okay I understand. And you so many of them will be using sanitizer

Respondent: sanitizer than water

Moderator: okay what about the guidelines. Do you have access to those guidelines been using on how to wash hands, are they accessible?

Respondent: there accessible we have been with all of them there. Okay

Probe: okay what about the other healthcare workers are they at their work station?

Respondent: they they have been put because some of the places just know any points am just trying to organize and pin more and to have all them. They were given all of them to all the stations

Moderator: umm now I would love to ask you few questions about stock out. Is there any period where you faced the stock out of gloves, mask, or you're not able to access sanitizer or some of the product that health work need

Respondent: at the beginning before there was sanitizer along the way somehow the problem of gloves came in but at the end of it we stocked all of them. When they became available we bought them in bulk so that we don't have to face it again. The same challenge again

Moderator: okay now I would love to know from that experience during that period, how did you manage? Let me say when you don't have glove when you need them? As a doctor,how were you doing it?

Respondent: it was hard. It was very very hard. We just had to make sure that now you know at times we say you people put on gloves but there other things that you know you have to what? You can even do without what gloves then you wash the hands immediately. Uh!

Probe: meaning if you see your patient without gloves you wash your hands

Respondent: immediately or you put the sanitizer next to you. After touching you do like this after touching you wash the hand like that.

Probe: what about the gloves, the mask?

Respondent: what about what?

Probe: what about the masks?

Respondent: the masks. The mask at times we have not faced it that much but at times use handchief.

Probe: ohh it was just a creation?

Respondent: it was just creating something

Probe: ohh you put handchief to cover your?

Respondent: to cover yourself. Double one.

Moderator: okay okay. Double one I like that initiative (laughter's). And how was it practically? Was it working?

Respondent: it was working though use something which is improvise it does not cover that space, it does not give you space that you can inhale air coming from the nose so it is some kind of that you have to use practically

Moderator: thank you so much for sharing your experience. Umm my last question will be about your recommendation. Umm throughout these three year we have seen what is Covid, what we can do and as focal person here so I think you may have some recommendation regarding this topic we are discussing. What can you say maybe to the policy makers, to other healthcare workers, to other IPC in charge like you? What recommendation can you give in regard to what we are discussing?

Respondent: Actually me, we have to continue teaching the community about corona. Me I know very well that these people they have not yet understood it well they only understood it by force because what because how they have seen it killing more people so we have to keep impacting more knowledge in them so that they understand it with any other related condition which is which comes like corona so that next time when the other thing comes they are not caught bad

And also ummm one thing have seen is when a new disease comes you have to use social media number one spread the news. As ministry of health it lagged behind a bit during the initial phase of corona so liers took advantage of it and went to social media and started putting lies there until when they almost defeated us. If the corona had not come became intensified not intensified on us you will not have gotten all this time to prove this people right or wrong but corona was real so we have to make so next time we have to use social media very well when it comes to fight diseases among the community people.

Probe: okay, what about the healthcare workers on how to use masks and gloves? Your recommendation, what do you think we can do better?

Respondent: Ummmm I think we need to improve the logistics because when something when like for me healthcare workers miss something then you bring like one or two carton and you disappear like for the next three months, it demoralizes them so just have to make sure that this thing moves constantly so that as we work we know that the only we are lacking is this one but not the essential one what we are going to use on patients.

Moderator: okay. I really appreciate. Thank you so much for sharing your knowledge and experience.

Respondent: you're welcome.

Probe: any last thing you would love to say?

Respondent: nothing much.

Moderator: okay thank you so much.

……….

TRANSCRIPT ROM

INTERVIEWER:

Introduction: Umm good afternoon once again. Today is 14th April, its 1:32 we are in Mbuya we are discussing about factors associated with compliance infection prevention and control measures among healthcare workers to minimize the risk of Covid-19 in Nakawa division. This interview has three parts. The first one we shall discuss about the barriers of usage of PPE and hand hygiene, the second them will focus on motivators of IPC compliance then lastly we shall ask you question about umm PPE stock out in case you experienced that during this Covid-19 pandemic. I will go to the first question which is about barriers of usage of PPE and hand hygiene.

Moderator: umm according to you what are some of the reasons that would hinder proper use of PPE among healthcare workers in your facility?

Respondent: umm the healthcare workers we have no problem in using the PPE because we know it is protection umm but the most ummm challenge that we find, there for buying we buy we use our monies to buy the facility don't like the facility does not umm give us the mask to use ummm basically that is it but we have no problem with using it

Probe: okay you mean that you provide for yourself?

Respondent: for the mask, yes but these other things like gloves what those ones they do provide

Moderator: umm maybe let me ask about the one you say they don't provide. I have heard some feedback from the people I talk to earlier, umm they report that they have difficulties in breathing when there putting on masks. Is it the same scenario around here?

Respondent: okay right now if am like the one am putting on, it discomforts the facility gave us but you cannot put it on today, I put it on because I didn't have money to buy these other one the Government one because those ones are okay umm apart from when you're moving it can be a little bit umm destructing, you sweat you do what but if at all you know it's a prevention, you continue putting it on.

Moderator: okay umm what about hand hygiene?

Respondent: that one is very crucial we do it every after seeing a patient or like after examining the patient, that one we have no problem.

Probe: so around so far I have seen that health workers are compliant in doing this?

Respondent: yes

Moderator: umm I have a particular question about umm the Covid-19 vaccination. Do you think that it has ummm maybe changed the behavior of healthcare workers about the use of how they wash their hands?

Respondent: yeah some for instance those ones who like were vaccinated fully they say I wll not put on like yesterday most of us went and got a third shot

Probe: third one

Respondent: yes a third one you know even if you don't put on a mask your okay with it eh it is okay even if you don't put on a mask you're okay. The virus will not get you.

Probe: but do they know that they are at the center of the community and wherever they are doing very important because they can transfer the infection from one person to another?

Respondent: umm what we do here we don't see a patient when he has no mask, yeah we do not they go and buy masks. At some point we found a challenge when they don't have money our patients are poor they have no money to buy masks you may end up just because for you, you have a protection you can't see a patient without a mask. But those one who don't put on a mask we don't do it when we have no patients around us but if at all you have a patient with you, you have to put on your mask

Moderator: what about the belief about the severity of Covid-19?

Respondent: ummm that one at first for the health workers maybe the community let me talk about the community, for the health workers we knew that it was there. Remember it came in three episodes, the first episode came and relaxed, umm that one we didn't experience it apart from those one that we had that died. The second one it hit very many people and we all complied people were putting on masks because most of us were infected as well because by then I was doing community work I got the virus and had just been vaccinated but now, after thereafter when people had died very many ehh people started putting on masks and they started observing social distancing so that is when we got to know that now there's Covid and is severe. One day I remember I went to the community I went to town, there was a taxi that I was going to board and around there I found a "mutembeeyi" and was serious you know calling people calling people and wasn't putting on a mask, I asked him why don't you put on a mask? He was like you tell me that first episode, you tell me who is Covid? And who is Covid? You tell me umm can I translate it to Luganda? like he asked me "*mbuulira oyo covid gwe yakuttira yali ani? Eh! Yatta Taata wa taata wo oba maama wa maamawo oba Jjajja wa jjajawo?"* like by then he wasn't understanding that Covid is real but after this second episode when people died those big ones, people became serious but now the I hear people are no longer putting on masks because the Government came out and say there's no more Covid so it is free to move without a mask, yes

Moderator: so do you think that we need to look at the way we form health workers because maybe from your point I understand that umm the Covid was serious but my information is not that (background noise)?

Respondent: it was but you know with Ugandans we take late to grasp some things we might say "***aaah banoonya byabwe"*** something like that. Umm but

Moderator: umm in your facility is there any approaches that you use to motivate healthcare workers so that they do the right things they know that this what am supposed to do? Put on masks, wear gloves then approaches that you use?

Respondent: we have SOPs to follow we also have a monthly meetings at where some of us have been reminded umm but with health workers we no task of doing it we know it. We know that we have to put on our mask

Probe: but I have heard a feedback from another person who would say that umm the compliance is kind of personal things because you should feel that its for your own protection

Respondent: ohh it is because now let me give you an example there's a doctor there but since I came here he only put on a mask on that day when he came he doesn't put on masks when you say that is also okay that one doesn't remove mask unless is eating or is in his form I don't know but even if is going on a market he puts on a mask and is fully vaccinated, yesterday he got a third shot

Probe: but he is always putting on a mask

Respondent: so it personal as you've said it may be.

Moderator: is there any support that you receive from families that motivate you? But you know from you that infection can shift from you, you go to the community

Respondent: no there's nothing.

Moderator: okay I will ask what to maybe to the last theme of this interview. Umm is there any period that you faced a stock out of the needed PPE? Because I remember you also mentioned that sometimes you have to buy for yourself

Respondent: yes since you have to buy them now like what will they I had to maneuver and go and ask this from the store because I didn't have and the N95 that I had when you put it on for long it let your ears pain so it need those lose ones like that one you've put on that one is okay but the N95 there those white ones that have tight rubbers so those ones are had when you put it on it will hurt your ears will hurt and you will feel a headache

Probe: but do you have are there days that whereby you had to use the several mask?

Respondent: ehhhh we use them we reuse them very many times like you buy like on a Monday I can buy three masks and that is for a full week, yes (laughter's) you buy, use like Monday to Tuesday or Monday to Wednesday, if your not safe a lot you put it on again. Your using and you feel like ah ah it is too much and you may even find people putting them on when they are dirty we reuse them my dear.

Probe: what about hand hygiene? Are there days you don't have like sanitizer for example?

Respondent: uh uh sanitizer we have. That one is always there at every point pharmacy, triage, there always there

Probe: those ones there always there

Respondent: yeah. Those ones but masks I think the institution does not buy.

Moderator: Now is there any recommendations that you love maybe to make for healthcare workers, other IPC in charges, maybe to the Government in case we are to face the same situation what can we do?

Respondent: for the Government, what I think now before the Covid, we used to have masks for every facility but now they it died off I think there positive now for me I think the Government it should give us surprise uh like take off the staff especially these NGOs to provide. Now like the health workers they wait for a month to end, you have loans and now here your bad eh! You have like ten thousand, masks are for one thousand, that is one mask and you have to use or getting maybe so if at all the Government should help us to give us supplies for free because we are its citizens.

Probe: what about trainings?

Respondent: trainings are very crucial, let them put the adverts on TV and may be people will listen to them and they will be like use Covid skills umm when you have t wash your hands you have to do this, social distancing ABCD and they have to maintain.

Then another thing, let those like police now like the taxi some taxis you don't go out when you dant have a mask.

Then another thing even us more so the healthcare workers and other people, it has to start from us

Probe: exactly

Respondent: it has to start from us. We have to know that this is our health it is our life when we don't guard it well, we are gone.

Moderator: yeah okay. Ummm we have reached the end of this interview thank you so much for sharing your experience and knowledge. Umm is there any last thing that you love to say?

Respondent: umm any other thing? Nothing.

Probe: nothing

Respondent: of course nothing

Moderator: Okay (laughter's) okay thank you so much. I appreciate, thank you so much

Respondent: you're welcome.

……….

<u>TRANSCRIPT NRH2</u>

INTERVIEWER:

Moderator: Good afternoon and good evening

Respondent: Good evening to you

INTRODUCTION. Thank you for being part of this interview. Today is 9th may and it is 06:09. Umm we are in Nakawa division we are discussing about factors associated with compliance to infection prevention and control measures among healthcare workers to minimize the risk of covid-19 infection in Nakawa- division. Umm this interview is going to have three themes. Umm we are going to discuss about barriers of usage of PPE, then we shall discuss about motivators of IPC compliance and lastly we shall discuss about PPE stock

out. Umm am going directly to ask the first question, umm which is about barriers for usage of PPE and hand hygiene.

Moderator: Umm according to you what are some of the reason that would hinder proper usage of PPE among healthcare workers in your department

Respondent: Umm repeat the question

Probe: The question is, what are some the reasons that you noticed or have experienced in what you seen during this covid-19 era. Umm what are some of the reasons that would hinder or what are some of the barriers for proper use of PPE among healthcare workers; I mean masking, gloves and all other Protective equipment?

Respondent: They went out of stock, the PPE and the maybe train like how to put on the PPE was one of the reasons

Probe: umm you mean that in your department where you work, do you experience umm stock out did you lack enough training, what was it?

Respondent: yeah some of us we were not trained well about those PPE because remember it was an emergency it came actually (laughter's) but for out of stock yeah we had some were out of stock like face masks used to get finished so you had to provide for yourself

Probe:: but have got also a feedback from people have been discussing with about the same subject and they reported that some scenario you the in charges you forget sometimes to order, is it true?

Respondent: noooo, it is not all about ordering, it was actually even the stores its already out of stock

Moderator: okay what about hand hygiene, do you find there some reasons for why health workers do not wash hands

Respondent: hand hygiene I think it is not so much a problem because we have water and soap, we have a sink we have water

Probe:: it was so much the water is was there throughout

Respondent: anyway, for the water it was not a problem. It was available and still available

Probe:: what about sanitizer?

Respondent: sanitizer, anyway somehow we used to get out of stock but you get like its all about your bill you go get you come, you go you don’t get, you have to figure around where you can get may be from the other ward and you share because we are a team

Probe:: and in the meantime how would you manage your healthcare workers, what will you tell them? How will they use to survive for all that time?

Respondent: umm some actually used their pocket money sometime and so they used to have according to the supply we use to have some

Moderator: okay. Umm now I wanted to know according to your own experience in what you see, is there any believe among healthcare workers about the severity of covid-19?

Respondent: yes we used to have the CMEs, we been told about the severity so we took it

Probe:: umm do they took it seriously?

Respondent: they took it because some of us brought in so we knew it is there yeah so they took it seriously it was serious actually

Probe: but taking it seriously did it affect anyhow how they put on mask or wash hands, use sanitizer?

Respondent: by then, it was actually eh… let me say it was a must yeah someone was not being actually reminded you all you knew that I have to put on mask to protect myself because it was serious

Moderator: and do you think that the vaccination changed their behavior? Are they still put it on compliance the way they used to do?

Respondent: no it is not right now. It is now actually some have relaxed a bit because of the vaccination (phone ringing in the background) it is actually they relaxed a bit.

Probe: but do they still have access to the same information do you still put on the same pressure or same motivation you used to do?

Respondent: anyway, we do but still that one are not forced to say that you put on a mask you have to leave someone but we we actually like we… put on ourselves so we remind ourselves how to put on a mask since it is now part of us.

Moderator: okay because I wanted to relate to feedback I also got someone told me that using PPE is it save behavior like you feel you're not

Respondent: actually it is now part of us. Yes so it now part of us

Moderator: okay. Umm I wanted to know is there any technique you use or you were using to make sure that healthcare workers put on PPEs, you wash hands

Respondent: yeah we have we put on PPE, put on a sanitizer we have our own sanitizer we have water availability of water yes and we get from the store so we get actually distribute these masks to each

Moderator: you distribute masks. Okay I want also to relate that to the feedback I got that someone use to truck what is giving to his health workers when they give you you sign to acknowledge that you have received PPE provided. did you have them also or you have them?

Respondent: we get from stores so we take like any other requisition we take to store then we being provided then we distribute them.

Moderator: do you have access to all the guidelines provided by the ministry of health?

Respondent: not really

Probe: okay what about the SOPs on the walls?

Respondent: here we have access to them

Moderator: okay have you noticed any preference among your healthcare workers between washing hands and sanitizing. Is there anything that you think they do more than the other?

Respondent: actually they do more a lot of sanitizing than hand washing.

Probe: Is there any reason with their choice?

Respondent: its actually order of the day even if there no covid so whoever your going to touch a patient you first go out you wash your hands and then after seeing a patient you come again you wash your hands. The order of the day. It is part of our routine work

Moderator: do you think that healthcare workers had enough training when accessed to PPEs?

Respondent: not all of them.

Probe:: is there any criteria that was using to access that it is this one and this one?

Respondent: they used to… go to those ones who are working in ICU, emergency like those ones yeah were more most in the training

Probe: you mean they were seeing those who were exposed?

Respondent: yes

Moderator: okay umm I wanted to shift to the last theme of this conversation about PPE stock out. Umm are there days you experienced a shortage of PPE?

Respondent: yes

Probe: umm can you share a little bit your experience about that?

Respondent: actually it was that period of two month covid we used to store when are out of stock then you have to leave these things either to go another ward to be helped to get a PPE or to reuse the available ones so that you can attend to the patient.

Probe: okay are there other measures that you would use to survive during that time?

Respondent: there was PPE we had to reuse the PPE to survive

Probe: okay are there period where you had to reuse the PPE,

Respondent: yes

Probe: okay, and what was your feeling when your reusing maybe it is also infected maybe?

Respondent: anyway you can't know but we were provided those ones so we first use them (laughter's)

Probe: okay I understand. Are there days where you had to for example buy for yourselves masks?

Respondent: yes we use to buy for ourselves, masks. You go home you don't have a mask and they did not provide yeah you had to buy.

Moderator: I will ask you maybe your recommendations but umm according to you and from your experience from what you have seen, what can be your recommendation to maybe the ministry of health, policy makers, other in charges and healthcare workers

Respondent: anyway what I can say people need more training even to avail enough PPE, masks, sanitizers. And all that

Probe: for healthcare workers?

Respondent: for healthcare workers training. They need more training.

Probe: any other thing you would like to add?

Respondent: maybe the they bring more PPEs.

Probe: like which PPE? You mean more masks, gloves or?

Respondent: gloves and any other. I think that's all

Moderator: that's all. Okay thank you so much for your time we really appreciate your time and knowledge, it was a good conversation

Respondent: your welcome.

……

TRANSCRIT: ROB

INTERVIEWER:

INTRODUCTION: umm thank you very much for allowing to have this interview with us. Today is 14th April 2022 and we are at Reach out- Mbuya. Umm the interview have four themes and we start with the first them. Umm like I have said the interview is going to be recorded so the first them is on the barriers of the usage of PPE and hand washing.

Moderator: so umm would like to know what are some of the reasons that would hinder proper use of PPE among health workers here at the facility?

Respondent: due to the stock because we are not always provided with the PPE so each health workers provides for him or herself so we might find one is able to get this medical ones and others are using cloth masks which are not good which are good to be used maybe when your seeing patients because of the infections you can get from patients can get from buyers

Probe: okay so apart from the fact that sometimes they get for themselves, is there any other reason you think that maybe they could not maybe use

Respondent: they always use masks as far as they are in front of clients. The fact that all except one is alone in a room, yeah.

Probe: how about during the high seasons of covid-19?

Respondent: there always used. Masks are always used

Probe: how about now that the pandemic is slowing doing. Are there reasons why you think they could not be using PPE?

Respondent: to the health workers, at this facility there always, they always use masks.

Moderator: Okay, okay and what are some of the reasons that could hinder proper high hand washing, hygiene among health workers

Respondent: here, I don't have reasons because we are always provided with sanitizer and we have it at the station outside. As you enter, sanitizer pot is there.

Probe: so you would say that all the health workers are provided with what to use.

Okay

Respondent: yes

Probe: but are you able to access all the necessary PPE whenever needed?

Respondent: no, as I said already the mask everyone provides so one can is able one may get for herself another one may fail or maybe reuse what you already used.

Moderator: so it's only the masks that they are not able to access

Respondent: but the gloves we have in fact others as I remained this side, the list only have gloves others we don't have.

Moderator: okay, and what is the basis for not having PPEs.is it like an administration thing as to why they don't provide?

Respondent: that one I don't know.

Probe: it's administrative?

Respondent: uh

Moderator: Okay. what about umm the belief that umm from the time Covid started in 2020, it was severe, did that in any way affect umm how the health workers complied with using PPE, putting on gloves in the different departments?

Respondent: not really (background noise) oba what can I say?

Probe: knowing that the dying you know there's that time when everyone was scared, everyone was contracting covid-19 did it in any way influence how the health workers use PPE?

Respondent: Yes because between that I think even during that period when the epidemic was high masks were used we had in fact people had our mask which is not enough then but epidemic was on

Moderator: okay how about now that we have vaccination campaign does it in any way affect how they comply with?

Respondent: however the health workers were immunized but they still put on the mask.

Probe: do they still sanitize like they sanitize when covid was at stake

Respondent: not really but again they do sanitize though not as they used to do during that time of the pandemic period

Moderator: okay, how about the need to protect others for example to protect other workmate, to protect the patient. Does it in any way influence how they comply with IPC usage?

Respondent: health education was done a day reminding people to put on their masks a and sanitize.

Moderator: okay, so what are some of the reasons that you think motivate the health workers to put on to comply with IPC? For example to wash their hands, to use sanitizer all the time, to wash you know every time they are going to see a client, to put on PPE. What motivates them?

Respondent: for that one, apart from what motivates the health worker may be the available sanitizer

Probe: if its there then they use it

Respondent: I think we have never got out of stock of sanitizer

Probe: how about gloves?

Respondent: gloves are used when you’re only doing procedure, yeah. If you have any procedure that need gloving that’s when you use the gloves.

Moderator: okay. Umm how about you know I know you mentioned that the health workers buy for themselves the masks does that motivate them in any way to use them? Or to buy for themselves. For example the people that work in the lab, what would motivate them to put on the masks every the other time?

Respondent: because we work, I mean we because you're working with the clients we don't know where they are coming from and some clients are reluctant in putting on masks and moving in taxis and some move long distance so definitely you have to because you love your life, you have to put on.

Probe: You have to put on, okay. How about for example let me you as a person as a health worker what would motivate you to wash your hands, to use sanitizer, put on a mask?

Respondent: I love my life.

Probe: you love your life (Laughter's). So that is the only reason as to why you put on a mask?

Respondent: and being an example to clients.

Moderator: But do the health facility provide health workers with IPC support for example those that have not trained, those that need different items to enforce IPC for clients

Respondents: I think we are all staying at facility umm by […]

Probe: apart from training, what else, what kind of support do you provide to health workers?

Respondent: that's all

Moderator: what are some of the approaches that you use to manage IPC compliance among health workers?

Respondent: we monitor ourselves, we remind ourselves everyday about the covid thing and the use of IPCs, the use of PPEs and hand washing, yeah.

Probe: okay. You also mentioned earlier that IDI comes and

Respondent: it mentors us every month.

Probe: And do you receive feedback from IDI about the support supervision that they do?

Respondent: there and then

Probe: how often do they do the audit?

Respondent: monthly

Moderator: okay. Umm the next section is hand washing station and sanitizer stations accessible to all the health workers

Respondent: yes of course we have hand washing especially at the gate as you come in and outside as you enter the rooms we also have a table that has a bottle of sanitizer

Moderator: as the IPC in charge, have you noticed preference among the health workers between hand washing and sanitizing? What do they prefer?

Respondent: they prefer sanitizing

Probe: what is the reason for the choice?

Respondent: I think sanitizing, where the sanitizer is easier accessing than to wash because we have the washing station just here a person coming from the other side coming to wash this way to was hands when there’s a sanitizer bottle, I think they prefer sanitizing

Moderator: And I know I asked this earlier but at every health workers station, are they able to access SOPs and IPC guidelines

Respondent: yes

Moderator: okay alright so we go to theme three which is about your PPE stock out and are there days when you experienced stock out or shortage of PPE the ones that are provided at the health facility?

Respondent: I think it could be few days in fact because where we are now, the main store is at headquarters and maybe the person ordering may order late and it could not they could not deliver immediately

Moderator: what are some of the measures or the adaptive measures or plan that you would have in case there's a (background noise) shortage stock out of PPE

Respondent: the in charge the person that is ordering to order in time not to wait until the sanitizer get done so she should order when she sees that maybe taking us like a week then she has to order

Moderator: and in cases where, how about in cases where the health worker is not in position to provide themselves or get their contact interaction with the patient. What measure are in place to mitigate that kind of exposure?

Respondent: health workers in fact they have to make sure that they have their masks, yeah.

Probe: and for those that use cloth masks, umm are there checkup or supervisions to see that those health worker is constantly not using the same mask without even or him washing it

Respondent: it is not there because we expect as health workers not to reuse the mask at least if you can use one today and wash and bring another one tomorrow. But probably here most of the

health workers they can buy for themselves medical masks, they rarely use cloth masks except the support of people

Moderator: are the days when the health workers do not for example umm hand wash or use sanitizer because it is out of stock?

Respondent: not really.

Moderator: okay umm what are some of the recommendation that you can give the facility managers to improve IPC implementation at the facility and compliance among the health workers?

Respondent: I think continuous health education to clients reminding them about the covid that we still have covid we have to live with it.

But also recommend health workers to do may be internal checks in themselves. You might find I don’t know not here but you might find that some health workers are reluctant about putting on the PPE so there should be some (background noise) internal checks

Probe: and how about (background noise) the health workers themselves what can you tell them so that they can improve the way they can comply with the different IPC measures?

Respondent: they can continue using the PPEs, the gloves.

Moderator: okay is there anything else that you would like to share with us. Anything that we have not mentioned, anything that probably missing but could be missing be important?

Respondent: yeah to you people that have come maybe for the survey maybe to take our voice that we need as health workers as a facility we need some provision of PPEs

Probe: which ones in particular?

Respondent: the masks

Moderator: okay alright thank you so much for sharing your knowledge and experience with us we really appreciate and thank you so much for your time.

Respondent: okay you're welcome.

……..

TRANSCRIPT: NRH3

INTERVIEWER:

INTRODUCTION. Umm good afternoon once again and thank you for being part of this interview. Today is 10th may and is 02:09 and we are in Nakawa. We are discussing about factors related to compliance with infection prevention and control measures among healthcare workers to minimize the risk of covid-19 in Nakawa division, Kampala Uganda. Umm this interview, will have umm mainly three themes. The first one (phone ringing) is about the barriers for usage of PPE and hand hygiene, then we shall discuss about motivators on IPC compliance and lastly we shall discuss about PPE stock out. The first question I will ask you is bout barriers.

Moderator: um what are some of the reasons that hinder the proper usage of PPE among healthcare workers in your facility?

Respondent: its availability was not stand there times when it was not there and the attitude people want them their at work they have to use them

The other issue was the work load was a bit heavy.

Probe: umm what about hand hygiene, sanitizing, washing hands?

Respondent: the same thing still availability. By that time the workload was like in my hospital still were many patients and the staff is the few staff who serve all those people so you find that they were overworked

Moderator: umm were you able to access all the PPE as required?

Respondent: umm, no.

Probe: can you expand more what was the issue?

Respondent: our supplies are not there they don't meet the whole demand. we are supplied …they use to supply us three times a year that is every three months or every four months but this supply was meant maybe for three or four months then you find the stock last for about one month when there no PPE and…

Moderator: okay. I would like maybe to shift the conversation maybe to what is commonly about the severity of covid-19. Umm has it affected in any way the healthcare workers could abide to this

Respondent: they were compliant to PPE and IPC because they didn't want to die they were seeing people dying.

Probe: what about the vaccination campaign has it changed in anyway in the way still comply to IPC?

Respondent: no our health workers who were vaccinated here to our hospital, they still see that this vaccination is proof that you won't go sick so have seen them comply with the readiness

Probe: okay is there any mechanism that you put in place to motivate them so that they still comply?

Respondent: our own mechanism is we have emphasized to them that IPC is for you not any other person. You prevent for yourself that is enough motivation.

Probe: and how is it working so far?

Respondent: it is working, its progress it is a continuous process we are getting better.

Moderator: and do you provide guidelines and SOPS to make them very visible so that they?

Respondent: we have the IPC material in the work station

Moderator: okay what about the fear of need to protect others. Have you got anything that in line with that that are motivate them still comply with IPC?

Respondent: the need to?

Probe: protect others you healthcare workers are at the center of the community they receive a lot of people

Respondent: Their first community is their families it is pertinent to them so they don't take anything back home.

Moderator: okay, okay. Umm I would like to ask you a question in regard to the experience you said sometime the supply is not enough. Umm what are some of the mechanism you use to make sure that you still practice and do the right work?

Respondent: we have we had people who were offering when we don't have supplies and their different company that came in and provided what was not around.

Moderator: umm what about the trainings because I have got the feedback that some trainings have been whereby they describe the training some will report to you that they will pick from your experience. How is the structure of the training from your facility?

Respondent: for now the training mainly the CME came in

Probe: and how is organized the CME? Sorry what is CME I may not know?

Respondent: continuous medical education

Probe: okay, how are they organized?

Respondent: at departmental level. Every department ideally should not actually wait so those meetings were not the CME with the content to IPC and training at that level.

Probe: okay were the topics been selected on the basis of any some ones recommendation or it a random topic?

Respondent: no we do need some assessment. We go to those wards and stand it for them, study it to and you say this is for every ward and their training need so we use this standing up to training up

Probe: so from the needs assessments you conducted such assessments

Moderator: okay, okay. I will go to the end about the recommendations. What are some of the recommendations that would make to the Government, other people in your position and healthcare workers in general?

Respondent: recommendation umm concerning IPC, it should be institutionalized in such away that the way money comes from drugs there should be money for IPC

Probe: okay is that all? Any other? For healthcare workers

Respondent: my message is still the same emphasize it for use as a health worker. Its not for any other person there's we are preventing you from policy something you should take serious

Moderator: okay. Thank you so much for your time, sharing your knowledge we really appreciate it.

………

<u>TRANSCRIPT K3</u>

INTERVIEWER:

Moderator: umm good morning once again?

Respondent: Good morning

Introduction: umm thank you for allowing us to be part of this interview. Today is 12th April and it is 11:41 and we are in Kiswa. Umm we are discussing about factors associated with compliance in IPC measures among health care workers. Umm this interview will cover mainly three themes; the first one will be about barriers of PPE usage, the second one will be about motivators and the last one will be umm will cover the experience in case you had umm PPE stock out.

Moderator: I will go direct to the first question; umm what could be some of the reasons that would hinder the usage of PPE among health care workers in your facility?

Respondent: umm thank you very much, I am by the names… when it comes to the barriers the usages of IPC materials for example masks and gloves it may be just the barriers, stock out barriers I think knowledge

Probe: the knowledge

Respondent: yeah the knowledge yeah so you find we have various levels of knowledge expert; there those who have stayed they did maybe seen of long long ago whereby we still in a very resource limited area so most times you improvise yeah sometimes you would you even work on a client without any glove and then you what get well and you don't even get the infection so they have that mentality maybe so I can say mentality of the healthcare workers towards the usage, their mindset, their yeah due to their experience from work like us we have been doing this me and many we come and Mukama n'ayamba yeah they don't care and that is the attitude, yeah their attitude.

And then secondly, what could I say what could be barrier of usage maybe availability yeah so if I have the resources available I can use them but when they are not available, could be one of those barriers for the usage of PPE for masks and gloves, yes

Probe: okay sometimes there not there

Respondent: yes

Moderator: okay umm thank you I will go to the second question which is about a hygiene umm in the series during this Covid pandemic, umm from what you've seen so far what are some of the reasons that hinder proper hygiene among healthcare workers in your facility?

Respondent: what is depending on what?

Probe: what is the barrier of proper hygiene to wash hands or sanitizer?

Respondent: I think there's no barrier it still comes back to one's personality. There's a saying that "charity begins from home" their mentality, the upbringing, or the hard to do something I think is just the person in somebody someone because you may come and do training, do seen different with all everything all even everything is available but they can fail to use them for example go to the factories, one time I was at the factory, they have PPE these things the helmet and what they use and the masks, they had these duty gloves and what have you but people don't put them on but why I asked them why are they doing so because the mentality of a person in them, or maybe they feel uncomfortable to use them then sometimes maybe they feel they can do without it only if so it's someone person. I don't know whether I have answered the question right

Moderator: yes am enjoying the conversation. Okay, Umm when you say they feel uncomfortable what do you mean?

Respondent: maybe because why do I say maybe they feel uncomfortable because sincerely they have provided the resources available but then what is making you not using or they may feel they are so busy to apply it's a wastage of time by putting on the mask putting it on of bringing on the glove so it goes back to the personality because mentality so charity begins from home. It is your own self sincerely am not going to give myself to the president that this person is not putting on glove he has done his duty to provide so it is my responsibility to make use of it but you think even make use so the person

Moderator: I will pick from what you've just explained and direct the question towards the …0553..for people umm do you think that health care workers believe that Covid is severe and can that influence the way they put on mask or not put on? It is the same thing for gloves?

Respondent: umm you see among healthcare workers until we have more dangerous diseases and even got it so you come if among the healthcare workers some of these pandemic that come up they are not new, why are they not new?; the most important thing if you know the mode of infection the positive agent, the rate of infection and then how it is spread that's why I may be here this a cholera…0549 I may hold even with their hands yes but they know the rate of infection is by oral but if they touch this patient now for example me I don't have gloves but I have to collect this sample and examine am going to wash hands yet my names are short and I will not have contact with my mouth my hand and my mouth wash my hand clean so to for among the healthcare workers the more dangerous disease is the Covid but so the world or the globe sees it they are more dangerous but we have suffered more dangerous diseases than Covid maybe its just exergulated so that alone can influence the usage of masks and gloves

Probe: so you mean they have seen worse things than Covid? so they okay it may influence the way they put on mask and gloves. Okay okay. Umm I will still expand on that what about the vaccination, do you think it was game changer in the way

Respondent: what do you mean by a game changer?

Probe: like since people are vaccinated they become more reluctant?

Respondent: no because remember this vaccination has been clear it just you prevent the severity of the infection it's not that its medicine that has been given its negative, no eh? Its like when you get BCG so never suffer from whooping cough no, it just prevent the severity that's why you find that among the health workers there some health workers who have never got vaccinated they have suffered and they go to their community there okay, yes.

Moderator: okay. What about the need to protect others? When you are at the centre of the community every person who needs help will come to you and if you touch someone is infected you may transmit the infection to the other person

Respondent: but there also more other infection other than Covid as you can transfer to the community other than Covid

Probe: exactly now

Respondent: when you people are surrounding Covid alone yet there more other dangerous infection; talk of TB, talk of Ebola, eh! talk of those parasite that you can touch you then, then apart from this one, why? See?

Moderator: okay so what can you do? Is there anything you do to motivate healthcare workers?

Respondent: yes though we motivate them since you know they say when you go to Rome, you must behave like Romans even though you may have your own myth and belief and what have you so how about my healthcare workers as I told you since it was a global cry out for infection control specifically for Covid, we do encourage health workers to use necessary to prevent and we do educational talks if I have seen it and then people refer to one on one talks we go to different departments, have them make sure by distributing the IPC materials for example this kind the guidelines and the they bring events for planned hand hygiene, self education and make sure they maintain standardized let me say okay generally that is it then they make the work environment clean, tidy before you begin work sometimes or disinfect your working area of course you learn that when your when your going to begin working disinfect your work area. Where your leaving, disinfect your area so its just a routine we do encourage them. Me and my team we do encourage them you find that our healthcare workers lets do the necessary to avoid infection of to avoid spreading the infection not only to our clients also to our family members so in case we do here you hear no issues and am her am going back home my children will like eh mummy mummy let me take your shoe inside from the rug so we do encourage our health care workers.

Moderator: okay the duration CME what does it mean?

Respondent: that is Continuous education

Probe: continuous education, okay how is it done?

Respondent: yeah you can just pick topics like earlier on I told you since IPC, IPC comprises of several some modules or sub topics like waste management, an hygiene that's WASH, now we have waste management, we have WASH, we have five S so the two topics so several times me and my team have picked topics to go and we educate we say that's like remind somebody on something they had in mind before so we keep on reminding ourselves in the CMEs

Moderator: okay and so far what is the feedback you're having, is it changing something?

Respondent: yeah it is changing something. By the way when you try to move around, we try our level to do waste education, we try our level to do best practice 5s you see these are the role of 5s you see where the bins are where they are supposed to be then the zoning we make sure when you come your work area looks neat. if you look around its not so badly off

Probe: it's well organized

Respondent: yeah its not so badly off because that's the 5s that is sorting, set, umm standardize, sustain and then shine.

Yeah and then you come to WASH, its generally hand hygiene and then our wash facilities. The WASH facilities that we have and then some washing facilities, we have toilets you can see when your entering the gate I hope you washed your hands

Probe: exactly

Respondent: yes you can see where there's those handing washing PPE and then what I mean the SOPs still there wash our hands the five moments of hand washing yeah so those kind of and then also pick talks like the what the hospital cloud infection you pick a topic how do you acquire hospital had infections those infection that have to go to hospital how are they got, how can you prevent them yeah those and of topics umm you can just

Probe: pick and then teach your staff

Respondent: yeah

Moderator: okay thank you so much. Umm I will move to the last section of this interview,

Respondent: yes please

Moderator: I would like to know is there anything any period during this pandemic whereby you faced a shortage of PPE?

Respondent: yes I remember in 2021 around May to July there were shortage of masks, yes and then during that time most of us got infection because of yeah there was shortage of masks and of course you know this world the people use of course the extra handling and then the challenge that I saw the experience that I saw, you find yourself you haven't put on the mask yeah I think one also hat gave a lot of close infection because sometimes you find you remover your mask its hanging on your neck while your removing your mask you take it away because you're supposed to have the SOP how to put on your mask yeah before you find someone is putting the mask is just here and there so maybe during that time there was shortage yeah

Moderator: and people were they using a mask more than sanitizer?

Respondent: yes. You get your one mask you may even use it for the whole week because you

Moderator: Okay what are some of the adaptive measures that people were using apart from reusing? Where they buying maybe?

Respondent: yes they were buying so if you have the money you can buy your masks you keep on changing but those that were available I remember even told the risk manager that apart from putting on myself if the mask couldn't come and they did bought for us some few and then we were like we use them sparingly. Since we were living in a resource limited and then also we don't give there also yourself there also to the Government, government yanyamba to yourself so also everything starts with you everything starts with me

Probe: okay like those who believe they need to protect themselves they will buy for themselves?

Respondent: yes they buy for themselves

Moderator: thank you so much for sharing your experience and knowledge. My last question will be about your recommendations. Do you have any recommendation that you may maybe for healthcare workers, the Government and people occupying top position like this in charges?

Respondent: yeah first of all before I go to recommendations, I mean before I have maybe like before I go to recommendations first get the challenges. You know at first Government I think yes there as the ministry of health of health they have the system of an IPC is there but it's not active. It became active when there's a pandemic, yeah so it beats my understanding why does people come up and start and they don't even involve themselves in implementing it you know so anything before implementing partner that come up that how this thing will be done like this because I think in other well developed countries some of these things are part and parcel of what of healthcare work but when it comes to my own country, it's like a by the way because I have been in I have been playing as an assistant but when I became again in that position being IPC focal person like am just being there an object yeah you don't know whom to report to, you don't know where the sort of yeah yes, um there's no line like from here you go here and visit like a straight line it's not there. It only comes then you see comes with hand washing program, eh! Sanitation for million you find IP they will becoming no it makes me feel it's an extra added work and surely bothering me you know so the Government has make me feel like it's part of me I feel like it's someone who has been employed and is using me and their money you see that yeah so there my recommendation is that the ministry of health should take their programs as really healthcare workers take it part of them IPC part of them a program to be part of them not let them first wait for the implementing partners to come and begin emphasizing and if you start forcing health workers implement something that is part of them yeah you see that. I hope you understood so that has been my preference yeah because all these things even one time I came we were they did have IPC money if you go okay 60 percent you will not even find one. Umm With me and my we came out with IPC money sorting our facility- Kiswa yeah fill implementation and control

money by putting up our facility but if you go around the ministry only will come up with a guideline or the manual if it is influenza, it will focus of influenza, if it is TB, they will focus on TB if it is Covid has come now there focusing on Covid, why? Let it be general, infection control and prevention. It is supposed to be part of healthcare you see that not because a pandemic has come that's when they begin exercising it no it doesn't work like that

Probe: so you believe they are not standard they keep changing when they focus on one thing they leave the other?

Respondent: thank you. You see they are focusing on Covid things like that. People are dying of TB, people are dying of multi drug infection, people are dying of malaria and when malaria is now going to go is when they is now there's a also malaria is when they are going to come, really??!! so something needs to be done.

Probe: so they need to standardize

Respondent: yeah we have for example as lab technician we have dangerous diseases that are might fight enough engines even more than Covid. Your saying there diseases just in a what in the mercury there there.

Moderator: okay they need something which is standard that can guide you in all the different diseases?

Respondent: yes so IPC since you're doing something on IPC later may be since you're doing master level, let them see the IPC is not going to be implemented by the Government of this country instead it is the implementing partners that come and force the health workers because for them they feel you se this is happening they don't make implementing partners to feel that this is what we are supposed this is part of the work

Probe: okay I understand your point

Respondent: because by that you understanding the challenges than *"ah tontawanya"* with your things *"kati bakuwadde kati oli wano onsumbuwa"* those kind of things harass the disease

Probe: they started

Respondent: yes

Moderator: okay I understand your point, what about the healthcare workers don't have anything

Respondent: the healthcare workers I feel we just need to sort out ourselves, our attitudes towards one our attitude towards infection prevention and control because as I told you in the beginning that

Probe: person

Respondent: that person in me that person in you and learned from I have never been trained on IPC this is what I learnt from class my dad am proud that my dad used to tell me that where do you apply what you learnt from class? Am proud my dad used to tell me where do you apply what you learnt from school? That takes me to apply knowledge ii want this IPC-infection control to the not even in class now here today am applying it so to have health workers when it comes to Infection Prevention and Control, its us not implementing partner to come and implement it, not the Governemtn you know they are normally occupying offices and eating his money lets prevent myself from getting infected, prevent my clients from getting more infection, and my family members in that way we shall what avoid certain diseases, yeah because of our practice as I told you in the safe there I have samples of mdr yeah if I was with a biased mind or not having infection control whatever in my mind, I would be "*kusaasanya"* everywhere infecting people around so it is the personality yeah because this whatever thing this IPC is not only applied in hospital even in your home yeah?

Probe: I remember you mentioned about those things

Respondent: yes back home you can practice this lady from infection for example hygiene, they used to teach us when you go from toilet first wash your hands you see that way that time when people coming from Somalia and they were coming with wash yeah. They used to tell us your

future lies in your hands I understand it yesterday when I was being trained on WASH then when your hands are clean then your future is bright and clean, do you know how? Your everything the mass language they say when you go to the toilet wash your hands, you would have washed off those jams, then wash your hands from corruption then you wash hands from what, then you wash your hands from safety then you wash your hands from so your future is what bright so you have your future lies in your hands. So if your future lies in your hands, then your hands there dirty then know that your future is also dirty

Moderator: (laughter's) I get it

Respondent: not just this normal washing of water and whatever, deep inside if my hands are clean then am free from that you free from kyafu then my future is what my future is clean, your future lies in your hands so do an hygiene.

Moderator: wow this is amazing (laughters's)

Respondent: you go back to that if possible and see from toilet, after eating, before eating wash your hands if you think so deep washing is hand hygiene is wash is beautiful thing so if you wash your hands from everything, your future is bright

Moderator: thank you so much musawo, I really appreciate your time and conversation, so your last word you would like to share

Respondent: now what do you want to share I think that's what I had to share and finally what I want is my word to the people out there or wherever you will share the truth is when infection prevention and control its not any other persons responsibility, it's your responsibility and other person yeah if you carry out that infection prevention and control its only prevented in a hospital or a community dispensary or anywhere but it is you, it is applied everywhere note it is not applied in specific area right from domestic to social places you find people dropping rubbish everywhere, people spitting everywhere you know their hygiene, someone is walking up (shouting) Jesus Christ eh you see that so I want my last word is that infection control and prevention it is everyone's responsibility and it is applied everywhere you are, observe that and everyone will be safe.

Moderator: thank you so much this is the end of this interview we appreciate

Respondent: thank you very much.

…….

<u>TRANSCRIPT NRH1</u>

INTERVIEWER:

Moderator: **umm good afternoon once again?**

Respondent: good afternoon

INTRODUCTION: umm than you for being part of this interview. Today is 9th may and it is 04:40pm and we are in Nakawa division. We are discussing factors associated with compliance to infection prevention and control measures among healthcare workers to minimize the risk of covid-19 infection n Nakawa division, kampala- Uganda.

Moderator: Umm I would like directly ask umm the first question related to our first theme which is about barrier of using PPE and hand hygiene. Umm according to you, umm what are the reasons that would hinder proper usage of PPE; mask and gloves among healthcare workers during that this covid-19 pandemic?

Respondent: One is lack of knowledge information. Some people have inadequate information about what these PPEs prevent (background noise).

Secondly, (background noise) secondly, Strategy you can have limited so you find that that time they requested us to put on two masks but before shortage we could not put on. You put on one then leave one for the next day like that. Yeah those are majorly the two problems that yeah.

Moderator: Maybe they were asking you to put on two mask. What was the main reason, did they give you any explanation and justification?

Respondent: About what?

Probe: to put on two mask. The one you were saying. Were there any given reason?

Respondent: reasons why they told us to put on two mask the reason was that umm the two mask are provide more protection compared to one.

Moderator: umm what about hand hygiene, washing hand, use of sanitizer. Umm is there any reason you found that is a barrier for health care workers are found to do it properly. what are some of them?

Respondent: yes. In this place, we also have shortage of sanitizer then also umm actually shortage because we could have little sanitizer then you have to economize it maybe you would use it and remain with nothing, that one is there.

Then also for the soap that time I hope you're you want me to refer to…

Probe: yeah all in that took place all this period

Respondent: At that time it was intense and not only but even now this hospital has got problems with water- shortage of water you know the storage system the water system first got some problems so on and off we have shortage of water. You have water two days, three days when you don't have water so providing water to wash hands was difficult even of you have soap, yeah

Probe: now n such scenario, how do you manovour?

Respondent: well the administs these people they had to clean we send them to bring some water to the outside tap because we don't have water, they can bring a jerry can of water put in our hand wash drums you know we use that one but not the same. It does not really does not help us to achieve the desired effect.

Moderator: okay, I would like to ask the belief that was how about the severity of covid-19? Do you think that healthcare workers in your department believe that covid was severe or not severe?

Respondent: at first you know this thing came in phases. At first people were just scared but they did not see the severity they did not experience. They just used to hear how it is killing people so literally everyone was scared so we used to put on those masks we used to you know but still they would put on mask and sometime people were moving with mask but when that wave came which killed people, there's a wave that came around November December, yeah that one we saw death here in the hospital, people were dying all the time, many of them would have left here here in this unit. We had to because this unit we run tests at the same time, also the space so we had to stop clinics we had to stop cases which are not emergency because we had to create room to receive patients covid patients so you would find that all that room is full of patients so in a day we could not fail to lose two there patients only in this unit and that one we moved for about one and a half months during that wave, yeah so that one scared us a lot people adhered, putting on mask, some of us would not even go and eat in the eating area s people were putting on the mask so when they assessed us they asked us to put on. Whoever would have got would what, put on even hand washing people struggled to see that they did it, yeah.

Moderator: okay. Now I will now I would want to go further and ask about your families. Were you able to go to your families with any given support regard to family

Respondent: during?

Probe: yeah that scenario

Respondent: we were just sleeping near for us we still use to come from home and work

Probe: you go home and you come back and work?

Respondent: yes only that you take caution. We leave the unit from here then when you get home you begin by going straight to the bathroom. Whatever you have, no one comes to welcome you. You take your things, you use your own wish and make sure you put your things in the right places when they are not contaminated. When you reach home the first thing you would do is to go to the bathroom remove what you're putting on and you put in the soap. That is what we use to do and then all the time, everyone used to have what- alcohol in small containers everyone at least used to have it in small containers that is how we did it but still the same umm our families still got infected because for you you might be working in the hospital but there are some people who move in the taxis they don't mind because they did see what was here so you can move with people in the taxi they don't have any mask, they cough so these people they go to their homes they got families and their children will get covid, children play together you get it and it comes back toyou.

Then also what we went through that time and health workers because it was first associated with us those people working in the hospital so you find that when you go home people don't want to so much to be near you because they are so much working on those patients so you must also be having some chances, yes

Probe: ohhhh

Respondent: yeah in the beginning that is for me that is what I experienced but as time went on, people realized that thing was find and it has come and it can affect any other person

Probe: but there should be more motivate your attitude also in the hospital that you want to be more conscious, you want to do the right thing?

Respondent: yes. Yes we tried we really tried to do the right thing here in the and also to give information to our clients, yes we tell them when you're coming here put on mask, no mask we can't attend to you like that. Those who come without relatives what we would give the mask

Moderator: you would give, wow that was good. Umm what about the vaccination? Do you think that it has affected the way healthcare workers around you, are they more adhering or have thy relaxed? Do you think they are still behaving the same way like last time?

Respondent: they are not behaving the same way, with coming of vaccine of course most of the people got vaccinated so basing on their knowledge, of course for them their knowledge told them that when you get vaccinated the risk of covid reduces it because of the information which ministry of health keep giving that even if your vaccinated you need to continue adhering so when we are serving these patients we still have to adhere up to now. You rarely find us giving service without mask. Actually when we go out, we feel out of place, when your in a taxi you feel out of place because your fearing because your putting on a mask. When your in a taxi you can be alone so they look at you as somebody who is stil behind but for us when we are here we are encouraged to put on mask but also us we know that it is important because we still get those patients, yes at least in a week in a month we get them

Probe: those who can't breath

Respondent: come with covid yes we give them but we know that the old they come with another condition so its is mixed up there but again we are able to see that this person must be having covid basing on the signs the patient is bringing that's what encourages us to give to the clients. When you're attending to some body for coughing what we still take care.

Moderator: yeah is there any technique that you use so that you make sure your health workers keep adhering stipulated in the guideline? Is there any techniques you use, is there any approach you use to motivate them?

Respondent: the technique okay sometime the challenge is their reducing with like many of them like in this unit, have a big population actually in the hospital maybe we might be having the biggest population with the staff together with the students we are very many other departments also but like maternity there combined though they are many but combined but here we are few and actually the emergencies so we… we did to pressure on the masks that time there was shortage so in this event they devised a register when we see a mask we give to someone a register sign here that you have received then we give twice a month, you receive at the beginning of the month then their in the middle depending on what we have so that one helped me to keep truck and who was not good then the one comfortable so that is how we used it. Yeah I think it was I think it was a technique.

Probe: that is a good one

Respondent: yeah and then also other protection, other PPEs the apron, we had to provide the place which was formerly not there like a box where we put the aprons so everyone need you're in a need of an apron you don't look for it, you just go and pick it from there. You help yourself.

Then also that time we provided gumboots yes. Just put them down there whoever needed to use would always got

Then even these dispensers were not there so with the coming of covid the hospital in partnership with other organizations made sure that we have those dispensers everywhere

Probe: yeah those ones have seen them

Respondent: so with even up to now we still receive from stores and make sure so long as your around. Every morning we fill them in and then also we make sure everybody this thing has got soap

Probe: so is there any person that was assigned in that room or still in an organization under the hospital, you have to fill the alcohol…?

Respondent: that is now

Probe: it's you who organized it?

Respondent: yes as me because I work with students and even staff allocate role that for you when yu come on duty you do cleaning, for you you fill in the soap, for you you fill in the other one yes but also our hygiene supervised the one monitors like that.

Moderator: okay do you have access to guidelines and SOPs forcovid-19?

Respondent: the guidelines we that time we got posters, even right now some are still there how to put off, how to use, use of sanitizer, yes like that now the also quality improvement so time came when they say that we don't want so many poster on the wall so it contradicts a bit so we have to remove those posters.

Probe: oh they were there you had to remove some

Respondent: yes we had to remove some of them because they said it was too much eh it was littering the walls it was not looking nice so they told us you clean up what that's what happened yeah but access like having a booklet which is easily available like on a working table we did not apart from those posters. We did not have them but we were given information we had some trainings, some orientations most of us especially even those who were in leadership positions they equipped them then also we gave the same information to the people who work with us but they gave mass communication, yeah

Moderator: okay they gave information. Umm now, I would like to go back on this last theme, which is about PPE stock out. Umm I heard you mentioning a period occurring where by you don't have all the PPE. What are some of the techniques that you use to manage those kind of situations?

Respondent: stock outs, so answer is sad already, for me I got a book even I have hat book it is on that table I got a book and I made sure according to the supply when they supply the masks I have a list all of them so when they supply the masks I come and make sure if they are few I give five five if they are more I give ten but I check and saw that so and so but you received you received at least this time and when am giving them I give with caution that you received this time we have received few so this time you might have to buy so

Probe: so you mean there times they buy for themselves?

Respondent: yes because there times we could run short completely because sometime we could reuse then you feel this one dirtened I have been with the other patient you through it away then you end up using most of your staff you were given then now before the next supply you must have to buy you know and keep yourself up to when we get the next supply, yes

Probe: are there those scenario where you had some of them reusing the same mask?

Respondent: yes very much. Because when you give somebody five masks andthen again you give after two weeks, these five masks if somebody doesn't buy another five remember sometimes you can put on one for duty then you feel your not good enough to move with that mask in the public you throw it away and put another one by the end of the week those five masks may not be enough so you go in and buy, yeah

Probe: what about hand hygiene. I heard you mentioning that situation of fetching water to department. umm is it practical you found it that practical when you don't have water does it work for you?

Respondent: it doesn't work. Actually it is we do it with a lot of complain you know water with what actually it is so painful because that same you don't have safe water these hygienist has to fetch water to clean the flow then she has to fetch water

Probe: for you

Respondent: yes and remember the toilets also may be having so somehow somewhere you have to miss out yes you find that you cannot give in you cannot wash all the time yes but now of course some people use sanitizer in between but we are informed that sanitizer has never been safe unless soap and water

Probe: exactly

Respondent: yes

Moderator: okay. Umm I will ask you finally what are some of your recommendation. What can you recommend to Government, ummm manager of hospitals and other people in your position?

Respondent: umm in as far as

Probe: hygiene issue is concerned. The discussed

Respondent: specifically covid or generally IPC?

Probe: general and covid which you have

Respondent: yes. Yes I would think what… using tha available resources and if the available resources are not enough because it is our health, so you cannot compromise infection prevention because you cannot spend yes so if what Government ha given is not enough for your own benefit you put in some money and make sure that you provide for yourself

Then secondly, economizing yes when you receive you try to ration it and see that you give what is enough for everyone not giving excess

Then of course sometimes we might have a stock out here when the stock is there in the stores so the best is to try to… you know to order in time put in you request in time such that you always have something yes

Then also for the stores sometimes they can be having some of these things and they don't inform me then for you you stay here not knowing that gloves is out of stock they also need to inform but at least as far as IPC is concerned, Jik we have already received please come for it, umm we have received sanitizer please come for it so that communication needs to be addressed, yeah.

So I think the other thing is we always get our supply from NMS so I think Government the hospital doesn't need to wait when there's need they should what put in some budget such that when we have a lag they can use that money to buy for us what we need instead of waiting for the supply from NMS which is not predictable yes.

Moderator: okay thank you so much for your time, we really appreciate it was a fruitful conversation

Respondent: thank you too

Probe: okay

………

TRANSCRIPT LLM2

Interviewer:

introduction: Umm Good afternoon once again, Umm today is 30th march and it is 10:25 and we are in Kinawataka, umm thank you for taking time to be part of this interview, umm we are discussing about umm factors related with compliance to infection prevention and control measures among other health care workers to minimize the risk of covid-19 infection

in Nakawa division. Umm I will go directly to our first section looking at the reasons as why health care workers umm

moderator: According to you what are the reasons that could hinder proper use of PPEs, mask and gloves among healthcare workers during this covid-19 pandemic?

Respondent: according to me umm some of the reasons umm some health workers they believe that they are working but sometimes they receive their salary at the end of the month so sometimes it may be very hard to buy the mask, the gloves if at all they are not provided so its not clear so for me I believe if at all the allowance they receive them in time

Probe: okay if at all, they provide the necessary requirements

Respondent: Actually most of the things are provided but not all so if possible like me personally I can afford I can provide myself sanitizers and mask and so on.

Moderator: I would move to the following question asking about an hygiene umm still the same umm what are the reason that could hinder proper hygiene among health care workers during this covid-19 pandemic?

Respondent: sometimes they can be cleaned up and maybe sometimes they have like there's time that comes the clients are very many here we need to work on the other, you need to work on the other so this time of going to work on clients can save someone life so some things you may go just need just gloves and you don't wash the hands and save someone's life. I think that one can work better

Then sometimes blood you know there times when you have an emergency when you have an emergency sometimes you do have that tide eh so when you use the gloves you just remove them discard then put on another one work on another person so things of sanitizing may not be caring so you end up just changing the what the gloves

Moderator: Okay I understand. Umm are you able to access PPE I mean mask and gloves whatever needy during this covi-19 pandemic in the facility?

Respondent: we were able because they were providing them but when covid-19 it came to place to infect like it was reduced a number of people who were having it has reduced, they were providing for ourselves so those basics not for the clinic but for your personal usage

Probe: okay you mean there's a period where by who was providing because I hear you mentioning that they were providing?

Respondent: umm the whole of the clinic because the managers was providing for us because he knew we are dealing with the clients and covid was high

Probe: okay but currently?

Respondent: we have to provide for ourselves

Moderator: now I would like to hear according to you, umm do you believe that I want to hear you point of view umm that the fear of covid okay people here that covd is severe motivate health workers to wear masks properly so that they reduce the risk?

Respondent: yeah me I believe when covid is severe we had to save our lives because the health workers are also like any other person outside that we all have the same type of blood just that we have that tension that we are health workers so when we get to know that covid is severe because some of us we are still young we needed that pressure so there's no way we shall survive if we are not taking serious if we are not taking it as serious so we have to take covid as serious so that we can achieve our goals

Moderator: okay so you mean that in your facility people believed that covid is severe?

Respondent: do

Probe: they do umm you think they take the proper attitude when they see someone is affected

Respondent: actually we are very serious on that. When we realize that you have the signs and symptoms we advise you on what to do

Probe: okay what about for you as a team

Respondent: for us umm actually let me tell the team flue was very common so flue was very common, we couldn't enter the clinic without mask so for us we had to put on a mask so we had

show people that covid is what severe so we had to show them since your putting on a masks there's water outside wash hands come in a mask and so on

Moderator: okay I understand umm what about the vaccination? Do you think that do you believe or what is your view about umm the vaccination campaign how has it influenced health care workers behave or attitude towards the use of mask and gloves?

Respondent: okay the case of the vaccination we health workers and people we have a different way we understand things okay me like me when am vaccinating I may not get exposed but in some cases you hear that when your vaccinated you get high chances of what of being re infected again so when am vaccinating me it doesn't stop me from putting on the mask and everything but we don't have the so long oba we don't have the same capacity of understanding. Others think that when am vaccinated it is well it is also of getting what infected

Probe: so from those people for what you have seen so far, from the facility what is their attitude?

Respondent: for us our attitude would be when we got the vaccine we do still put on our mask because you believe you can get infected okay you may not only get covid but any other infection so we still see it necessary

Moderator: okay I understand, what about the need to protect others you know at some point it was an important topic they say you help healthcare workers your always in contact with these cases and you have to pay attention so that you don't affect the person. Do you think that from what you've seen so far that has motivated healthcare workers to be compliant?

Respondent: yeah it has been because umm how do we do it because for us as the health workers here when I think something new oba when I get to our heart beat I come around to the ground and so I ask people have you heard about this about macron so that we keep update so in case of anything we health workers because these people outside they see us because if we don't do the right thing they will say laba musawo is not putting on why me so we keep on updating ourselves and if our clients come around still we keep updating them

Moderator: okay thank you. I would like to go to the second scheme of this interview which is about the motivators of IP compliance, what can motivate the healthcare workers. Umm according to you, what are those reasons that may motivate health care workers to feel that put on proper mask, wash hands so that because covid is there what are those reasons

Respondent: okay please some of the reasons that can motivate health workers one; if at all they can provide for them because there's if at all they can say we are providing for them the mask, the sanitizer, that one can motivate someone because you have been provided for everything is to balance so someone I like I use my one thousand that I use to take me for breakfast and I buy mask how like covid working, how can I wait so sometimes there motivated because if at all they are getting the necessary thing is very easy for them and sometimes you know we are not motivated only by but when you sit see someone dying you motivate someone by saying you know I should put on a mask I should sanitize so that I feel I stay alive

Then the other thing we are motivated as basawo because like other people see from us so when we do the rest will do to so that's what motivates us as basawo to motivate because there other leaders see for them so that one can also motivate them

Probe: when you're sharing am enjoying

Respondent: then another thing when we get information like from other friends you know because basawo is a bigger thing so when you see the TV outside the country people are dying seriously because for us here people were dying but outside it was worse so that one also used to motivate basawo to put the mask and do the rest

Then another thing that can motivate to put on a mask or gloves because you cannot work on a client without those things you can't really go to test someone covid without putting on those things because you really know that it challenging because is a client wanted to a musawo your looking to the thing so we look different so that there's that motivation even the client will say eh musawo is good and possibly those can, can also motivate the rest are motivated

Then another thing motivation we should put sensitization updating them that one can also motivate people because I may not know something but if you come and tell me I learn and that one motivate me

Moderator: okay thank you so much for sharing your experience and knowledge. I would I want to ask still in the same angle, you as a manager and the team you work with do you provide them with may be support, motivate them, talk to them to the right thing does it happen

Respondent: yeah it does so happen because first of all I motivate not as manager because I am supposed because am also

Probe: part of the team

Respondent: yeah so when you have sensitization like as in within team of course we deliver masks, sanitizer, and refreshment yeah because when you refresh yourself you know even you brain is always updated yeah why not you're providing refreshment you're providing the necessary things in the clinic and shows that the manager is scaring because he shows you what is on the ground because for other people they don't even have time of meeting there always busy busy busy but if at all you come on ground you tell them this what should we do this is what is going on really your motivating those people

Moderator: And according to you, between hand hygiene using sanitizer or washing hand water and soap, which one healthcare workers in your facility prefer?

Respondent: us we prefer washing hand

Probe: okay what is the reason?

Respondent: the reason because our washing thing it is on the entry point but sanitizer someone may not see it because we put it somewhere where there's an open place but If someone is entering this it is the first thing you come across so you would feel as me a health worker I will feel pity if I don't wash my hands after working but it will be difficult for me to sanitize and I feel really inside my hands are clean at least if I wash them even me I know that I have washed my hands more than when I use sanitizer

Probe: meaning you prefer washing hand you feel it is more efficient

Respondent: when I wash my hands I really feel I have washed because I really do wash everywhere my figure but when you use sanitizer I just smear and for me I prefer washing hands

Moderator: okay for you, you prefer washing hands, do you have access to guidelines for covi-19 that show you what is the right thing to do and the procedures

Respondent: we do have access

Probe: you have access? But do you use them?

Respondent: because they are at the main entry so everyone when enters looks how do I wash my hands, how do I put on the mask, how do I do everything

Probe: okay so even healthcare workers they learn from there

Respondent: we are the most efficient ones because we do daily

Moderator: What about where you dispose gloves and masks that you have used, do you have proper location and bins

Respondent: yeah we have proper disposal because for gloves we have where to put gloves we have where we put our shirt, we have where we put our so we have a proper disposal within our clinic to avoid infecting people and infecting our health workers.

Moderator: I will go to the last section of this interview umm which is about the experience may be that you faced during this covi19 pandemic. Is there anything you didn't have enough PPE

Respondent: yeah that thing so happened it so happened because there's during a time during covid there's a thing we had nga it is costly nga there expensive so nga you have to get when you put on a glove and you check yourself nga it is not a glove (laughter's) you have to use it another person because it is costly buying it it is costly so you have to utilize the one you have so when we used to see that gloves are not nga you really see that it is possible to use to another person nga you can improvise and you use it for another person

Then for the case of sanitizer, because for us our clinic we use to make ourselves sanitizer but there's that time covid was too much nga when your sanitizing you just do one so you reached a time and sanitizer got done but before you do not even make another one so there's that time our sanitizer was done because we were busy the time we could use to make sanitizer because it was necessary because we had that line to work on so we were trying to improvise with the hand wash

Then masking they promised to bring us the mask the Government unfortunately it didn't bring. Some of us we didn't receive okay but we used to improvise we use to buy the cotton this mask the cloth you cant afford the other one you buy that one and you wash so that's how that time it was difficult but unfortunately we went through that

Moderator: okay I would love to hear the process you shared about making your sanitizer umm is it that is it a disinfectant that you mix with sanitizer or its alcohol?

Respondent: we used to make the real alcohol sanitizer. Yeah the real one not this not alcohol taking but this one the one for sanitizer we use to make for ourselves

Moderator: okay thank you so much musawo umm we have reached the end of this conversation but the last question is always about the recommendations. What recommendations do you have for healthcare workers in your facility in others in broader level may be to the Government to people who decide on what to do?

Respondent: okay my recommendation however much we are like covid has moved but people should continue putting on the mask, continue sanitizing, do the necessary as for your personal health because this is okay we are talking about covid but its not only about covid, health workers there very many severe things even more than covid so if you get an update update your friends, your clients, put sensitization as it comes show people what to do umm if you can afford something do something better for the people, yeah

Then for the case of the Government ha…the Government should take care of the people and should improvise more of services of the health worker because if you go to other cases very many things are missing because for the case of the Government it reach a time nga there no service yeah

time come nga they don’t have drugs though you work for them they are like you go and buy but something for government why by the time someone goes to the government he doesn’t have money for the clinic so why should I go to the Government and they tell me go and buy so such things should be looked it because they are coming they have to take care of us. So that’s all from me and thank you so much

Moderator: okay thank you so much for sharing the information, it has been good and this is the end of the interview.

……

TRANSCRIPT KC2

INTERVIEWER:

Introduction: umm thank you once again for being part of this interview, it is 10:39 on 30th march. Umm thank you very much for being part of this interview. We are discussing about factors associated with compliance among health workers and this interview is mainly focus in the usage of PPEs, gloves and masks and umm other items for health care workers.

Moderator: umm the first question we have for the respondent, is around barriers for usage of PPE and hygiene. Umm I would like to ask you know some of the barriers that healthcare workers faced or are still facing during this pandemic for the usage of PPE. Umm I would like to ask you what are the reasons that could hinder proper usage of PPEs among health workers during this covid?

Respondent: umm what could hinder proper usage of PPEs, gloves and masks, okay one of them is if your putting on masks people find it challenging you know people have that mindset that when you put on a mask you don’t breath well and then also the people when have some complication so when they put on mask they don’t feel comfortable then also gloves umm gloves putting on gloves it was seen like it consumes some people see that it consumes time and sometime when you put on gloves you don’t wash your hands because their some kind of gloves that have that white umm can we call it chemicals then

Probe: powder

Respondent: Powder so people think that it consumes time and it makes even spoil because every time if you have like ten patients you may be forced to put on like ten gloves or six or five so people think like it makes you waste gloves, especially during this period of covid so people find it challenging in away that you use a lot of gloves by doing that you will spend a lot of money

Then for the issue of masks when you put on sometime you don’t feel comfortable even your breath even your voice won’t come out well

Probe: you can’t talk to your patients

Respondent: you can’t talk to patient freely that’s why people find it a challenge and also buying masks also, it’s not easy because buy it you spend which not all facilities have that activity to buy masks regularly

Probe: do you mean that in your scenario for example you buy masks for yourselves

Respondent: yeah and at our facility here we buy masks for ourselves in most cases we buy masks we even buy gloves umm I can say most of the PPEs we buy, umm I remember once in a while the ministry helps us with some few once in a while

Moderator: okay now I will go to the other umm points we are focusing on during this interview about hygiene umm according to you umm what is the reason that may hinder health workers to properly sanitize or to wash hands

Respondent: okay you see umm what washing hands people are not used to washing hands some people they are not used to washing hands so changing somebody’s mindset also is also not easy mm the mindset is one thing then also like find that even access to water may also hinder somebody from washing and say that ah is ably like I can even do without washing hands

Probe: okay what about sanitizing?

Respondent: umm sanitizing umm when you say more of mindset it was mindset and also umm not ably all bodies have sanitizer because umm you need to buy when the ones the Government has given gets used up you need to buy and yet they have money for sanitizers

Moderator: okay umm now what was your approach during this covid-19 and then like when you have may be to face umm many patients at once in case you had a scenario

Respondent: many patients of covid?

Probe: no not many patients for covid or during these different waves I think did you have any period where you had many patients like you need to treat more than usual

Respondent: yeah we had a lot of signs of cough and flue so what we do would find a suitable place or would make sure we put on masks and the few gloves we had we put on then we also make sure that the clients who come put on they also put on masks depending you better come when you have a mask so if a patient comes without a mask you go and buy then they put it on they come then we attend to them. And we attend to only one at a time.

Probe: but were you able now to change properly masks in that situation?

Respondent: when there many my dear its not easy to change you may even forget because you're looking at time you had a patient in bad condition you forget and you feel like I think let me just put on this persons gloves but again by doing that you may also be risking but most people forget and people are not used to that is that

Moderator: okay do you what is your point about the belief on the severity of covid-19 in regard to the usage of PPE and hand hygiene

Respondent: what is my point?

Probe: in other wards do you think that people take it seriously so that they can use properly masks and wash hands? Health care workers

Respondent: ummm yeah people you see people have this mindset umm I want to give an example when covid came people found were just a way of just threatening people but people took it it was not serious it was not even in Uganda at least we have a scenario where somebody had all the signs and symptoms here and unfortunately the person passed away that's is the time when I saw everybody was putting on masks, serious issue now masks, gloves. That person patient used to come here but the way his breath everything changed but we wanted better go to Mulago and he refused unfortunately he died but following the information we had later he was died of covid that little bit of it we lucked that testing umm kits and also let me say that service of handling a covid

patient apart from the referred but even the one we referred could not accept and a man died, that is the time I said I should put on mask the whole community even the community when that man died like this everyone here everybody you see will put on a mask but now then after people had gone back to that normal thing especially now I think this works you see people have mindset that when they get the vaccine they are safe so now issues of masks, gloves are not

Moderator: exactly that was my second point I wanted to ask you about the vaccine if you think it has changed peoples attitude

Respondent: it has changed because people now have that mindset that okay umm since I have got the vaccine umm am safe because with what saying that when you get the vaccine even if you get covid, you don't die you don't suffer severe condition so it has changed peoples mindset the people are free and some of them are even said they are getting the vaccine not even some but most of them

Moderator: But during your community of health care workers do you have say attitude or you would say that in your community people have that attitude

Respondent: people have that mindset even health workers that since I have got the vaccine am safe from covid even gloves you put on if you have that blood, body fluids, but covid now is now history because people only have that mindset that when they got the vaccine they are safe you don't die you don't suffer that severe state

Moderator: okay, umm do you like am sure after work you have to go home and meet your family umm do you think because you know that we have that saying that covid is still coming and they suggest people to keep those umm protective measures so that in case that was to happen they are still safe and protect others because you know we have had different waves of different and you see someone severe the vaccine did not respond as we expected umm now in your community of health care workers in your facility, do you think that the fear because your always in contact with people and you need to protect your family that can motivate you to do you think it can have an impact so that you protect them?

Respondent: yeah it has then what I can say is that there health workers let me say when you are like us, possibly like us working we have sanitizer we have the gloves so always before we go to our families we make sure we wash hands, sanitize we do that and also make sure that even our

families get those ones that are in that age they get the vaccine then we make sure we all get the vaccine but we wash hands I don't know you see washing hands and sanitizing needs you to get used to it but it's not easy

Probe: but why it's not easy?

Respondent: it's not easy in a way that people look at it like ah! now when I don't wash my hands where did what did covid and these have to take me and people have that mindset that even when I pray God can save me you see, all that comes across

Moderator: I would move now to the second part of the interview, which is about umm motivators what can motivate healthcare workers to compile IPC measures. Umm do you umm do you receive support like do you receive support of healthcare workers regarding to IPC motivation do the right thing

Respondent: supporting ministry or supporting the facility?

Probe: the management supporting your facility.

Respondent: um there's no support,

Probe: okay there's no motivation?

Respondent: even I want to show them to the management or ministry, there's no motivation and that's what most of us what I can tell you is not there either from the minisrtry or may be whether from the management it is not there there's no motivation

Probe: there's nothing they do to motivate you?

Respondent: no apart from buying but also buying is not easy because health facilities its not easy to maintain if your to follow buy PPEs all the time its not easy because the money which is there is not enough taking all that.

Moderator: okay I understand. Umm what about your family did they tell you musawo do the right thing, doctor do the right thing

Respondent: yeah they tell you they do they do they do.

Probe: any other way they motivate you?

Respondent: umm I don't think. What I can say what they do is from home they just keep monitoring me or telling me that oh covid is there so as we handle those patients and then care because we don't want to hear that the person from that facility so they keep on to keep you alert telling you that you that you need to be watchful and taking care

Moderator: Okay, umm in your facility do you have access to umm cans washing instruments all the time with water and soap?

Respondent: we have

Probe: then it is always provided like sanitizers

Respondent: yeah sanitizers we have sanitizers even have water like for us we have borehole Askari can just bring water and just bring put some soap, then we have sanitizers, yes we have them

Probe: but now between hand sanitizer and washing hands according to you what is more practical and feasible for the healthcare workers in your health facility

Respondent: you see sanitizer if you look at it like it saves time you just spray umm moves there ah your good to go but again washing hands you need to open water, apply soap move your hands in all the sides see so it is a process but with sanitizer you just work smear your good to go so most people prefer sanitizer to water because of that. People wash hands majorly may be when they are going to eat, is when you see most people wash their hands with water

Moderator: okay umm I will ask you another question, it's about timelines in the SOPs. Umm do you have access to those guidelines or SOPs on how to wash hands, the proper use of PPE?

Respondent: umm the guidelines we have we have that

Probe: you have the poster?

Respondent: the posters we have them but sometimes you see when the patient comes you may not follow them you can just say because we have been using but of course when you get some emergency, you need to follow all the steps that's the thing and that's what I found most health workers do, yes.

Probe: but when it is an emergency situation?

Respondent: somebody need to follow all the steps you see somebody only sanitizing putting on gloves

Moderator: okay I understand the point. Umm but in that scenario you may be knowing that they are not doing the right thing but they have the knowledge

Respondent: Umm you see, you people have knowledge but putting it into practice is of course another thirst that's what I can say so they have to make or define of putting it into first and also people's mindset, people look at it like people forget let me say people forget using a word under some condition people forget but people have the knowledge but they can forget also.

Probe: but you said they did first they wash

Respondent: that's true

Moderator: okay umm do you have umm places where you can dispose of waste using masking, gloves

Respondent: we have and those people yeah we have them where we can dispose and yeah. Then also like these others, uniforms, there's a company that comes and picks and it take dispose them where they should be disposed

Moderator: umm you know that those ones are potential carriers also for infections umm do you think that umm it reminds you as health workers that you have to dispose them properly, because they are potential danger they can have covid and any other infections

Respondent: yeah

Probe: or you think they have forgotten

Respondent: probably, proper disposal of waste is very important because in the point of carrier to infection which I think covid yeah when I think you need to ensure proper disposal of waste gloves masks because they can carry infections

Moderator: okay I will love to ask you in another area, are there periods in this covid-19 pandemic where by you faced shortages of PPEs we are discussing about? And if so what was the approach given that time? I remember sharing experience of you having many patients sometimes, you have to attend, so what was your experience?

Respondent: your asking me

Probe: you do have enough masks, you don't have you have to treat many patients

Respondent: well we used to have masks, gloves but now we were forced to sanitize especially masks, I mean the gloves sometimes to use them more than it is required like masks a patient comes and seriously presenting signs of covid, you first put on a mask we use the same mask disposable then gloves were forced to use one pair of gloves for like three four patients because there was shortage so that one happened

Moderator: okay umm now to finish what can be your recommendation to umm facility let me start from there, the managers, the ministry what is your recommendation or the message you want to share with them if you look at the challenge of PPE, what health care workers are facing so that they know what to do in case we face the same situation of covid-19

Respondent: umm what I can say; one the supervisors or let me say the board and the ministry you should find out time to take up the leads to health centers and sit down with the health centers health workers ask them what are they lacking, that is one.

Supervise them see, me what have seen is the board is relaxed, the ministry also relaxed then they could bring something there's supervising let me say. I will also make colorful people health workers what are you lacking and make sure that they provide health workers with the basics the PPEs, gloves, masks, all the time they also provide funds that can be used to buy some basics committed at the health facilities

But also have some motivation something to motivate them to work.

Then also trainings are required because some health worker don't even know what is taking place they luck ministry programs which would help them to widen or have them what is required. Honestly trainings are good if they can have trainings, supervise, provide basics which are used at the facility thn I would see a change.

Then also increase the number of health workers that one is also will also help because if health workers are many you don't work on pressure because if you have so many patients and health workers are few, then your intention you want to save this client see this client so increase on the

number of clients the number of health workers then motivate them. Then how to change them, interact with them that challenges are helps it helps to share and see how we can move forward but mostly do you know that most managers don't have grace they don't have to see that health workers need this

Moderator: okay thank you very much for sharing your experience around this topic, this is the end of this interview, this is the end

Respondent: your welcome

Moderator: okay

………..

<u>TRANSCRIPT B4</u>

INTERVIEWER:

INTRODUCTION Umm thank u so much for allowing to attend this interview with us. Today is the 22nd April 2022 and we are at Benedict Medical Centre. Ummm like I have said this interview is going to be recorded and the interview has four themes and we start with the first theme which is about the barriers for PPE use and hand hygiene.

Moderator: umm (background noise) what are some of the factors that hinder the PPE use among health workers?

Respondent: Ummm first of all there not there and if they're there they are scarce. (Background noise)

The other factor is that some people may not be well acquainted of how to be used umm so the knowledge of how to use (Background noise) may not be well spread out to the health workers

Probe: okay thank you but what are some of the factors or the reasons that would hinder health workers from practicing proper hand hygiene to the different health stations especially during the current pandemic?

Respondent: of course one first of all the fear of Covid.

Also secondly, you said what would hinder?

Probe: what would hinder them from practicing proper hand hygiene?

Respondent: exactly umm lack of litter gentle from water itself, lack of litter gentle of alcoholic based solutions call it sanitizer

Moderator: are you and the health workers able to access PPEs whenever needed?

Respondent: it's a yes

Probe: it's a yes. Okay

Moderator: umm are there cases when the health workers are not able to access that PPE?

Respondent: Just like I said if they are not they run out because you have to change every time you go there so in terms of the adequacy may not be there, so yes its possible.

Moderator: has the severity of Covid-19 in anyway affected the IPC compliance among health workers in the facility?

Respondent: yes I think it's the umm the what can I say the fatigue the stress of range so you may not actually do the right thing in terms of IPC for example downing or hand washing they skip certain steps.

Probe: but given the fact that everyone had fear of covi-19 dying the next time umm wouldn't that in any way influence the health worker from saying that I need to wash my hands, I need to put onw what am supposed to?

Respondent: they did what am trying to say is that run out as observed during that time. Umm in terms of supply chain was a problem especially for the PPEs as you said umm because of therapy

so there was ban out really associated with that so those lapses within the fatigue because they were really in large numbers in a way I can take it all.

Moderator: okay, umm what about the fact that we are in the Covid campaign has it in any way affected how health workers implement or practice?

Respondent: yeah positively. There more we stick to hand wash and observing the guidelines.

Moderator: what are some of the argument of for IPC compliance given that were are in a campaign right now and everyone is advised to be vaccinated for Covid-19?

Respondent: of course the vaccine most people taken it some people skipped it so acceptability let me say acceptability to the vaccine was very strong based on (Background noise) can I call it the adverse effects or side effects or other complications itself associated with the vaccine so there was that issues that's what remained.

Moderator: okay we all know that health workers are at a higher risk of being exposed to Covid-19, right from the beginning even up to now. Has the need to protect others also influenced how they used the PPE?

Respondent: I think so

Probe: to what extent?

Respondent: umm first of all following the guideline and ensuring I think part of it is regular hand washing or sanitizing umm and then being to suspected cases in isolation, what I noted that because of fear of health workers taking a portage home it was kind umm you put them in a situation where there always a little more keen I may say in terms of observing distance, and really the guidelines if you make any contact let say to any suspected cases that is observed but they said at times you may confront the person and at times you may not follow for example you have large numbers few PPEs so you find yourself actually repeating the same PPE umm yeah. I don't know whether that answers the question

Moderator: it does, thank you but what kind of support does the health facility provide to health workers in regard to IPC

Respondent: to provide like what in terms of prevention, PPE, what they need in terms of soap or detergent that is provided or sanitizer is provide them of course depending here and there on IPC training

Probe: okay umm are these trainings on job trainings, mentorships or they receive the trainings and the mentorship from IPC or any other department?

Respondent: all. From the facility and then the other external IPC

Probe: okay are these mentorships extended to all the health workers in the facility?

Respondent: yeah

Moderator: okay what are some of the approaches that the health facility uses to manage the different IPC systems that are in place?

Respondent: ummm what can I say (Background noise) of course as you said we try to scrutinize and see what we can implement but we may not implement everything that is available what can I say for example they call it the aerosol I don't know whether you have heard about it but it's more of (Background noise) it was hard for us to cope that spraying the rooms so we decided to improvise with Jik what we are able as the patient is discharged as opposed to what other people would do.

Moderator: is there any support that is provided to the family of health workers or extended to them?

Respondent: yes unless there's a have signs and symptoms otherwise as you said the staff or the health workers show the signs and symptoms actually the tests rapid test

Moderator: have there been difference between hand washing and sanitizing among health workers in general Covid thing

Respondent: mainly sanitizing

Probe: why the preference to sanitizing?

Respondent: its easier and the points are you can control it because opposed to water that are morally stagnant and at specific points

Moderator: umm among health workers on your notice what have been some of the adaptive measures you see that a health worker has employed in the absence of PPE or sanitizer or any other

Respondent: they have used aprons, they have used umm requested for at least two pairs of uniform and at times they take a shower before they go home, yes

Moderator: okay that's good. Umm are there cases where they had to buy for themselves certain kinds of PPEs?

Respondent: yeah

Moderator: but are there cases when they had to reuse?

Respondent: yes

Moderator: okay alright. Um what are some of the recommendations you give or suggest for the facility managers, public health advocacy in line of the implementation of IPC and

Respondent: I will only say one thing, umm IPC should not only come during Covid even if it's one area that is aggregated I think everyone will be sorted why because even before sanitizers let me try to recall were only available in more less restricted areas ICU and those things of theatre it

was even in that thing of Outpatient and or wards which I think now should be something let me say it’s a norm for general hospital set up not just hospital departments.

Probe: you also mentioned that there may be reluctance and may be ignorance among some health workers on how to use and the proper way they use the PPEs. Why do you think?

Respondent: umm (heavy wind blowing) I think the person routinely engage in PPE use and also support supervision so but I think most important our duty is support supervision or even focus from the higher level the ministry level to ensure that IPC is not just what is pushing down but something regularly check

Moderator: okay alright thank you so much. Is there anything else in line with IPC or generally that you would like to share with us or maybe we may left out

Respondent: yeah I think it’s eh… (heavy wind blowing) based on operation in a sense that for example if something is mild so those who do not get vaccinated are able to come up with that remedy or the national immunity after we come for it I think that’s an area they need to address on yeah because new contacts can come up anytime and those that may not have full induction propagated to assertion

Moderator: thank you so much. This is the end of the interview, we really appreciate the time and knowledge.

Respondent: okay.

………

<u>TRANSCRIPT B2</u>

Moderator: Umm good afternoon once again?

Respondent: Good afternoon.

INTRODUCTION: today is 10th may and it is 02:56 and we are in Nakawa. We are discussing about factors associated with compliance and infection control measures among healthcare workers to minimize the risk of covid-19 in Nakawa division Kampala- Uganda. This discussion will turn around three themes. The first one is about the barrier for the use of PPE and hand hygiene, that second one is about motivators of IPC compliance and lastly we shall discuss about PPE stock out. I would like to ask the first question which is about barrier for usage of PPE and hand hygiene.

Moderator: according to you, what are the some of the reason that would hinder proper usage of PPE among healthcare workers in your facility during the covid-19 pandemic?

Respondent: thank you so much for that question, I think the barrier the biggest why people are not using these PPE is attitude because people for example our facility we have everything in place but once in a while you find that someone is not sanitizing, someone is not wearing gloves, someone is not washing their hands. Otherwise there aware and everything is provided so it is attitude

Probe: okay attitude. So you are able to access the PPE when needed?

Respondent: when needed that's true we can access them

Probe: okay, umm is there any other reason that you found to be a barrier apart from attitude?

Respondent: maybe at times maybe the work load because sometimes maybe people feel like if they have this line here if I have to what to work if I have eight client I don't have to do what they continue with their work maybe the work load

Moderator: okay, what about the belief about the severity of the infection of covid-19. Has it affected in any way the behavior of healthcare workers in the facility?

Respondent: I have to see that during the first will be just talked about covid in every begin, people would not say about covid so that's why am like I don't know whether there's ignorance then, they never believed that covid is there not until the second umm wave of covid when people started seeing people dying and that's when people started saying they were like covid is serious and during the second wave actually you find that people were washing their hands they were very keen and strict eh doing everything as per no one would be moving without a mask and even compelled his mask with the quality you know but as these phase umm phase one, phase three people are no longer dying. During this period especially during this very period where now when they are now I think now it's the record that they have discharged the last patient from Mulago we are going back from where we came from. Umm the masks had become uncomfortable, they coped using the PPE that were provided. after that announcement that we no longer have covid cases, I think people are giving up and this business of having all the health workers vaccinated people feel have that confidence that at least they have that protection, they were vaccinated so they can wear their masks once in a while in a taxi.

Moderator: once in a while, okay. Of course that was my second question I wanted to ask you the vaccination campaign has affected in anyway their behavior

Respondent: I think it has. After the vaccination people have this feeling that okay am protected even if am to get covid I will get that mild covid

Moderator: okay. Let me shift to the motivators of IPC compliance. Umm do healthcare workers in your facility have access to the guidelines and SOPS?

Respondent: I think so. I can say affirmatively say that they have access because you can have them whenever you need them. You know we have the gloves so it's a matter of reposting this is what I need and it will be provided.

And the times would make sure like for sanitizer we have someone who checks on people's containers and see that

Probe: okay so is it a person that was assigned or just took that

Respondent: we had consented and also sometimes you need to tell them that you know though some people forget like the technicians they don't want to move out of their room if you don't give them they will not yet they need them most.

Probe: because feedback I also receive from the other person was following you, we were discussing the role of filling all the bottles around the hospital and sanitizer. S you feel it works?

Respondent: I feel it works because that is ensuring that the sanitizer is there no complaint otherwise the health workers may be complaining that is one thing about the health workers. When it is there, they may not use it but when it's not there, they will make noise, they will make noise

Probe: what about the training of IPC?

Respondent: were trained especially when covid was started in the first phase we had training series of trainings. People were coming here to train us. We got trainings even from the school of public health I remember it was mr. manze who came from that place. We had people from the ministry it was doctor am actually forgetting the name but it was a doctor so actually we were trained

Moderator: okay and how was those trainings organized? Was umm all the health care workers did they have access to those trainings

Respondent: actually all had, anyway the beauty with us we are few when the training is there we just call all of them and you have the training.

Moderator: okay, okay. Umm let me now ask about umm the preference between hand washing and sanitizing. Do you have any pressure that healthcare workers have a tendency of preferring one to the other?

Respondent: you know I think yes they prefer sanitizing to hand washing

Probe: do you have any reason to that

Respondent: you know its easy to use your like yeah a time I feel like sanitizing its easier just sanitize but now as far as I have a sink outside it requires me to first stepping out getting to a sink which is not n my office.

Moderator: okay, okay, umm now let me shift to the last section of this interview about stock out. Is there any period you experienced stock out of PPE?

Respondent: I don't remember since we started covid because we have been supplied by donors, KCCA, ministry so things have been coming.

Probe: so in your scenario you never?

Respondent: in my whatever in my facility I will not talk for other facilities but in Bukoto actually we had enough gloves, we had enough sanitizer we had enough Jik so liquid soap and bar soap used to come so would not even be like we experienced a stock out but one thing, no

Probe: no then secret behind that success of that.

Respondent: maybe because of covid I think so many people were scared because we were hearing people dying abroad and people were very much concerned about health workers who are so we ended up getting support you know people came in donated, us we benefited from so many things because those are the things we are still using even now like Jik, we got powdered Jik, we got liquid Jik and it is a lot still we still g=have those cans because experiencing stock out, no.

Probe: masks, gloves?

Respondent: even they are still there we get boxes of gloves

Probe: they give or they gave enough

Respondent: yeah a lot was donated and cases procured and we still have. It's a matter of putting in our request if we don't have them we are to blame because they respond.

Moderator: umm what about hand hygiene washing hands,

Respondent: washing hands it has also catered for

Probe: you didn't get any challenges in that area?

Respondent: umm no. just when we even had that constructed the hand washing never used to be there

Probe: ohh it's a new?

Respondent: it's a new thing that was constructed by GIZ and wash program we never used to have that one we used to have we had a lot of tap you see them on the compound they were many they were enough you get tap every corner each entry under council we were umm taken care of.

Probe: and there still working and your still using up to now

Respondent: now that we have that one some we are not using them maybe in some areas like in where we brought others inside the is still working

Moderator: I will ask you now to give some recommendations from what you seen and experience shared, umm what are some of the recommendations?

Respondent: the recommendation for me what I will think maybe every infectious condition like an outbreak it shoud be given due what can I say due cause like people should come in and support like they did for covid so many organizations came in to support and people could come when

people coming like you know, some sales are working some sugar, you know? That motivates people to come to work like transport was catered for it motivated them to come to work because they were being picked from home you know they bring you to work they take you back home so they still like you know the transport was given we were like if we can be given some of those vehicles to cater for the health workers transport because we don't have accommodation here it will help a lot.

Transportation of the health workers

Probe: okay what about the attitude of healthcare workers as you mentioned? What can we do?

Respondent: changes a process we need to keep on talking to them and but the stretch is not that much and people have this confidence they have been vaccinated so this business of telling them wear a mask every now and then but hand washing they are still doing but now we are having a challenge of masks. You know at times often the only challenge will be some things have the mask but the quality if the mask becomes a problem. The mask are there you see (laughter) the loops are too low we discard. Eh you have to tear them I will have you know a material would be okay it's not so bad but are they are very disturbing but the masks are there. Both the cloth and these disposable.

And the other thing was N(% people are not used to it they were like it was giving them whatever it was affecting their ears so much they will be like it pains when you wear N95 for a long time. Of course truth to be told we are giving all types of a masks we had N95, is it KN95, these disposable the two so they had issues with N95 and these KN95 so like there not comfortable when you put them on. May not be attitude but the comfort also is an issue

Probe: what about hand hygiene you said that its some how people improved. Is there anything we can do to maintain that good behavior?

Respondent: you know the issue is you keep on talking and reminding people that you know as much covid is declining but their other effects we can get that can come in.

Umm these posters I have just received that should be off it removed our posters if you have poster everywhere when someone reads, it's a reminder. Your reminded of what to do so we need those IPC IEC materials concerning hand washing, they can help

Moderator: okay thank you so much for sharing knowledge and experience. Any last thing you would love to say about this topic?

Respondent: about covid. Need support and people to continue supporting the team. The team that is doing the work even the motivation.

Facilitation to make people happy. It may not be monitoring we have like transport. Transport was a very very big motivator to the staff. People who were picked from home and there taken back you know? The issues of late coming were solved, issues of absenteeism were also no excuse, when someone is at home, someone will tell you am sick today you don't have anywhere to prove but when a vehicle is ther someone will actually see that when am sick or not,

Probe: they will take you.

Respondent: they take you and even these over early living a like when people can come here and reply that two three is not there because you know the vehicle will be picking at five so it concerns. Even late coming is reduced because we picked you from home you are not waiting for a taxi because a taxi would go pick all those issues were solved. I think that would

Moderator: okay, thank you so much this is the end of the interview it was a very good conversation.
